# A Novel Cooperative Behavior Assay in Rats Reveals Distinct Social Coordination Strategies of Increasing Complexity and Social Reciprocity Deficits in a Model of Fragile X Syndrome

**DOI:** 10.1523/JNEUROSCI.0143-26.2026

**Published:** 2026-05-13

**Authors:** Ashutosh Shukla, Edward L. Rivera, John H. Bladon, Shantanu P. Jadhav

**Affiliations:** ^1^Department of Psychology, Brandeis University, Waltham, Massachusetts 02453; ^2^Neuroscience Program, Brandeis University, Waltham, Massachusetts 02453; ^3^Volen Center for Complex Systems, Brandeis University, Waltham, Massachusetts 02453

**Keywords:** autism, cooperation, Fragile X Syndrome, prediction, social behavior, theory of mind

## Abstract

Cooperative behavior, the ability of individuals to coordinate their actions toward shared goals, is fundamental to survival and social success across species. However, the mechanisms supporting cooperation and disruptions leading to social deficits in neurodevelopmental disorders such as autism spectrum disorder remain poorly understood. To address these questions, we developed a novel cooperation task in which rat pairs had to visit matching reward wells on paired spatial mazes under deterministic and probabilistic reward contingencies, utilizing dyads of littermate wild-type (WT) and *Fmr1* knock-out (*Fmr1*) rats, a model of Fragile X Syndrome. Both WT and *Fmr1* male rat pairs exhibited dynamic turn-taking with mixed leader–follower behavior for cooperation; however, WT pairs achieved significantly greater cooperation success than *Fmr1* pairs. WT and *Fmr1* pairs both successfully utilized a simple follower-tracking-leader strategy, resulting in slower asynchronous transitions between reward wells, with *Fmr1* pairs more reliant on this reactive strategy. WT rat pairs also exhibited a more efficient and flexible predictive cooperation strategy, requiring anticipation and coordination of optimal transition patterns with peers, resulting in faster synchronous transitions. *Fmr1* rats were unable to utilize this more efficient social reciprocity strategy of higher complexity, leading to deficits in adapting to the probabilistic reward condition and in cooperative behavior. These findings reveal distinct strategies of varying complexity for cooperation, demonstrating the existence of a complex predictive social reciprocity strategy in WT rats and its disruption in a rat autism model, providing a foundation for investigating mechanisms underlying social interactions with relevance to neurodevelopmental disorders.

## Significance Statement

Successful cooperation requires monitoring others' actions and flexibly adjusting self-behavior, yet mechanisms underlying cooperation and associated deficits in neurodevelopmental disorders such as autism remain unclear. Using a novel spatial cooperation task in rats, we identify hierarchical strategies that support cooperative behavior—a simpler follower-tracking-leader reactive strategy and a more complex reciprocity-based predictive strategy that enabled synchronized coordination of actions with peers. While both WT and *Fmr1* rats exhibited the slower reactive strategy, only WT rats flexibly adopted more complex predictive strategies for efficient cooperation. *Fmr1* rats thus showed selective disruption in social reciprocity due to impaired ability to predict and synchronize with partner actions. These findings provide a novel framework for dissecting the behavioral and neural mechanisms underlying social cooperation.

## Introduction

Cooperation is essential for survival in social species, enabling collective action, resource sharing, and learning (Axelrod and Hamilton, 1981; Wilson, 2000; Krause and Ruxton, 2002; Nowak, 2006). These complex skills develop through observation and coordination within social groups, characterizing decision-making in natural environments (Giraldeau and Caraco, 2018). Individuals constantly integrate information about their own goals with the actions of others in continuous reciprocal interactions, navigating interdependent and dynamic social contexts.

Successful cooperative interactions involve observing or predicting partners' actions, adjusting self-behavior in real time, and coordinating toward shared goals. These features make cooperation particularly informative for understanding the mechanisms of dynamic social decision-making. Moreover, impairments in social communication and flexible interaction are central characteristics of autism spectrum disorders (Boucher, 1977; Soderstrom et al., 2002), especially, Fragile X Syndrome (Budimirovic et al., 2006; Salcedo-Arellano et al., 2020), highlighting cooperation as a tractable framework for studying the biological basis of social impairments.

Investigating behavioral principles and strategies for social cooperation can provide key insights into social cognition and inform intervention strategies. However, existing paradigms for studying cooperative behavior remain limited. Many rely on turn-based games or simplified tasks with fixed choice structures (Gardner et al., 1984; Avital et al., 2016; Wood et al., 2016; Delmas et al., 2019; Schweinfurth and Taborsky, 2020; Jiang et al., 2021, 2026; Shin and Ko, 2021; Zhang et al., 2025), which constrain opportunities for dynamic coordination, partner tracking, and reciprocal engagement. Developing ethologically grounded experimental designs that preserve the richness and flexibility of real-world social interactions is critical for advancing our understanding of cooperative decision-making and its neural underpinnings.

Rats can be an ideal rodent model for these investigations due to their advanced socio-cognitive abilities and natural prosocial behaviors, including reciprocity, group foraging, and coordinated decision-making (Nagy et al., 2020, 2024; Schweinfurth, 2020). Rat foraging behavior in spatial contexts has been extensively studied since Tolman’s early experiments (Tolman, 1948), and rats also tend to show more complex social play behavior such as hide-and-seek (Reinhold et al., 2019; Kaufmann et al., 2022). In contrast, mice have a limited social repertoire and demonstrate simple social behaviors (Crowcroft, 1966; Nadler et al., 2004; Weber et al., 2017; Cum et al., 2024; Vogt et al., 2024; Jiang et al., 2026). Importantly, several prior studies have attempted to probe cooperative behaviors in rats (Daniel, 1942; Rosenbaum and Epley, 1971; Schuster, 2002; Avital et al., 2016), underscoring their capacity for socially motivated interactions. Rats’ cognitive abilities, the rich repertoire of social behaviors and the availability of monogenic rat models with targeted gene knockouts such as *Fmr1* and *Nlgn3* (Hamilton et al., 2014; Till et al., 2015; Saxena et al., 2018; Asiminas et al., 2019; Klibaite et al., 2025), can provide a framework for investigating mechanisms underlying social coordination.

Here, we introduce a novel spatial cooperation task for rat dyads navigating two opposing W-mazes, in which animals must coordinate with a social partner to obtain food rewards by synchronizing visits to complementary maze reward locations, even under probabilistic reward conditions. This task takes advantage of rats' natural spatial foraging behavior for rewards and enables intentional, real-time coordination. We show that efficient cooperation in this task can be accomplished by reciprocal coordination of choice strategies with peers and through real-time monitoring using peer-directed attention. In addition to an asynchronous, follower-tracking-leader reactive strategy, wild-type (WT) littermate pairs also employed a more efficient predictive coordination strategy to execute synchronized cooperation, especially under probabilistic reward contingencies, similar to nonhuman primates and humans (Konvalinka et al., 2010; Pecenka and Keller, 2011; Haroush and Williams, 2015; Skewes et al., 2015; Meisner et al., 2025). Fragile X knock-out littermate rats (*Fmr1*) were deficient in this complex social reciprocity strategy. By quantifying coordination strategies and decision dynamics in both wild-type and *Fmr1* knock-out rats, our findings provide a foundation for understanding the behavioral principles and neural bases of cooperation and how these processes may be disrupted in neurodevelopmental disorders.

## Materials and Methods

### Animals

Male LE-*Fmr1^em2Mcwi^* rats (*Rattus norvegicus*), hereafter referred to as *Fmr1* rats, and their WT littermates were used in this study. We use male rats in this study since Fragile X is a X-linked syndrome with less severe impairments reported in females than males in humans, and homozygous mutations in females are extremely rare (Bartholomay et al., 2019; Vafaeie et al., 2021; future studies will examine heterozygous females). A total of 23 WT and 24 *Fmr1* rats were included, with all subjects aged 3–5 months at the start of behavioral training. Pairs of rats comprising WT-WT, *Fmr1*-*Fmr1*, and WT-*Fmr1* were used in cooperative behavior assays, with group sizes kept to a minimum number (*n* = 5 pairs each), given the longitudinal profile of the experiment, and as reported in previous studies (Tan and Hackenberg, 2016; Reinhold et al., 2019). Rats were obtained from breeding colonies and were group housed until they were ready for experiments (3–5 months). Prior to starting the experiments (1–2 weeks before start) and until completion of experiments, rats were single housed under a 12 h light/dark cycle in a climate-controlled room (temperature, 20–25°C; humidity, 40–70%). Importantly, the period of single housing was kept consistent across genotype groups. Food and water were provided *ad libitum*, except during behavioral training, when rats were maintained at ∼85–90% of their free-feeding weight to ensure their motivation in the task.

### Ethical considerations

All experimental procedures were approved by the Institutional Animal Care and Use Committee (IACUC) at Brandeis University (#21001, #24001-A) and conformed to the National Institutes of Health (NIH) guidelines for the care and use of laboratory animals.

### Methods

#### Behavioral apparatus

Rats were trained on elevated W-shaped mazes (80 × 80 cm) with ∼7-cm-wide track sections, each consisting of three arms radiating from a central junction (Shin et al., 2019; Tang et al., 2023). A reward well was positioned at the end of each arm, equipped with an infrared (IR) beam-break sensor for nose-poke detection and automated milk reward delivery. Two mazes were placed in parallel and separated by a transparent acrylic divider, allowing visual and olfactory access while minimizing physical contact between the animals. An overhead color CCD camera (30 fps) was used to record sessions to monitor positions of animals on the mazes.

During behavioral shaping, rats were initially trained on elevated linear tracks (∼80 cm long) equipped with the same IR sensors and reward delivery system to establish nose-poke behavior and familiarize animals with automated reward collection prior to cooperative task training. Behavioral apparatus was controlled using a SpikeGadgets data acquisition system (SpikeGadgets).

### Experimental design and statistical analysis

#### Pre-training and behavioral shaping (Fig. 1)

Prior to training on the cooperative learning task, rats underwent a structured pretraining regimen on linear tracks (80 cm × 7 cm), designed to familiarize them with reward-seeking behavior via nose-poking in designated reward wells. Initially, each rat was trained individually to spatially alternate between the two ends of the linear track to obtain rewards. This phase encouraged the development of goal-directed navigation and spatial alternation, fundamental skills necessary for subsequent cooperative tasks.

In addition to spatial alternation, rats were trained to execute and maintain nose-pokes within the reward wells with a stepwise increasing hold duration, ranging from 0.2 up to 2 s. This incremental hold requirement ensured that the animals learned to sustain their response, a critical component for successful reward acquisition in the cooperative learning paradigm where timing and persistence are essential.

Upon demonstrating proficiency in spatial alternation and nose-poke holding on the linear track, rats progressed to the social phase of behavioral shaping. In this phase, pairs of rats were placed on parallel linear tracks separated by a transparent, perforated acrylic barrier allowing visual, auditory, and olfactory communication while preventing physical contact (Avital et al., 2016). Both rats were required to navigate to the same end of their respective tracks and perform simultaneous nose-pokes in the reward wells to obtain rewards. This phase was designed to promote coordinated action and social interaction, facilitating the development of cooperative behaviors critical for the main cooperative learning task.

This gradual shaping protocol ensured that rats were not only trained in individual task components but also conditioned to synchronize their behavior with a partner, thereby providing a robust foundation for assessing cooperative learning in the primary experimental paradigm.

#### Cooperative learning task (Figs. 1, 2)

**Figure 1.**
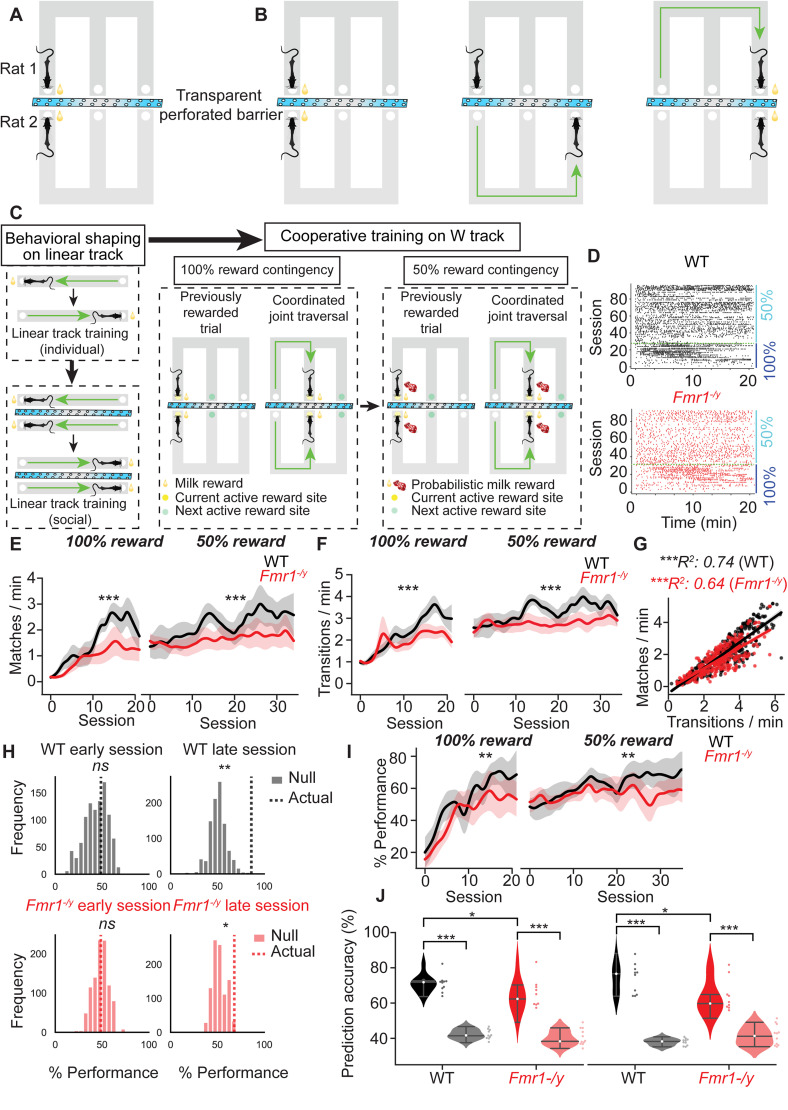
Cooperative learning task and coordination efficiency over training. ***A***, Experimental setup. A pair of rats run on complementary W-mazes separated by a transparent perforated barrier to seek milk rewards. ***B***, Example of a successful cooperative event. Both rats occupy complementary wells on their respective mazes (spatial cooperation) and poke simultaneously together in their respective wells (temporal cooperation) to collect milk reward (left). Following a successful match event, Rat 1 moves to another well on the maze (middle). A successful cooperative event occurs if Rat 2 chooses to go to the complementary reward well on its maze and poke simultaneously with Rat 1 in the respective reward wells (right). ***C***, Experimental design. Left: Rats were trained to run back and forth on a linear track alone (individual phase) and with another rat (social cooperation phase). Right: Following proficiency in both the individual and social phases on the linear tracks, rats were trained on the social cooperative learning paradigm on 2 reward contingencies in succession: 100% reward contingency (where every cooperative event was rewarded), followed by 50% reward contingency (where cooperative events were rewarded probabilistically only 50% of the time). ***D***, Raster plot showing match events for an example WT (left) and *Fmr1^−/y^* (right) rat pair. Dotted horizontal green line denotes transition from 100% reward to 50% reward contingency. ***E***, Match rates for WT and *Fmr1^−/y^* pairs for 100% (left) and 50% (right) reward contingencies across behavior sessions. 100%: Two-way RM ANOVA (genotype: *F*_(1,168)_ = 26.8338, *p* = 6.289 × 10^−7^, session: *F*_(20,168)_ = 6.0003, *p* = 9.243 × 10^−12^, genotype × session: *F*_(20,168)_ = 1.2808, *p* = 0.198). 50%: Two-way RM ANOVA (genotype: *F*_(1,280)_ = 27.0576, *p* = 3.82 × 10^−7^, session: *F*_(34,280)_ = 0.8733, *p* = 0.6735, genotype × session: *F*_(34,280)_ = 0.3742, *p* > 0.9995). * denotes genotype-specific differences, ***p < 0.001. Shaded error bars denote SEM. ***F***, Transition rates for WT and *Fmr1^−/y^* rats under 100% (left) and 50% (right) reward contingencies. 100%: Two-way RM ANOVA (genotype: *F*_(1,378)_ = 11.7127, *p* = 0.0006886, session: *F*_(20,378)_ = 6.9567, *p* < 2.2 × 10^−16^, genotype × session: *F*_(20,378)_ = 1.5533, *p* = 0.0613132). 50%: Two-way RM ANOVA (genotype: *F*_(1,630)_ = 38.3755, *p* = 1.054 × 10^−9^, session: *F*_(34,630)_ = 1.2923, *p* = 0.1265, genotype × session: *F*_(34,630)_ = 0.7761, *p* = 0.8170). ***p < 0.001. ***G***, Linear regression between cooperative match event rates and transition rates for 50% reward condition. WT: *β* = 0.79, *R*^2^ = 0.74, *p* = 3.696 × 10^−104^, *n* = 35 sessions; *Fmr1^−/y^*: *β* = 0.64, *R*^2^ = 0.44, *p* = 1.907 × 10^−79^. ***H***, Top*:* WT pair: Observed versus shuffled coordination performance across training. Null distributions of coordination performance for a representative WT pair during an early session (100% contingency, left) and late (50% contingency, right) session are shown. Vertical dashed lines indicate observed values. Null distributions were generated from 1,000 circular time-shift permutations of one animal’s behavioral sequence, preserving individual choice patterns while disrupting inter-animal timing. Observed performance did not exceed chance in early sessions (ns) but significantly surpassed the null distribution in late sessions (*p* < 0.01), indicating learned coordination. Bottom*:* *Fmr1*^–/y^ pair: Observed versus permuted coordination performance across training. Same analysis for a representative *Fmr1*^–/y^ pair. Observed coordination did not differ from chance in early sessions (ns) but showed a modest, yet significant increase above the null distribution in late sessions (*p* < 0.05), suggesting limited improvement in cooperative timing with training. ***I***, Performance metrics for WT and *Fmr1^−/y^* pairs in 100% (left) and 50% (right) reward contingencies shows significant impairment in *Fmr1^−/y^* pairs compared with WT pairs. 100%: Two-way RM ANOVA (genotype: *F*_(1,168)_ = 7.4888, *p* = 0.006876, session: *F*_(20,168)_ = 4.3806, *p* = 3.823 × 10^−8^, genotype × session: *F*_(20,168)_ = 0.4842, *p* = 0.969754). 50%: Two-way RM ANOVA (genotype: *F*_(1,280)_ = 7.5354, *p* = 0.006441, session: *F*_(34,280)_ = 0.9385, *p* = 0.570405, genotype × session: *F*_(34,280)_ = 0.3594, *p* = 0.999687). ** denote genotype-specific differences, **p < 0.01. ***J***, Prediction accuracy of a multinomial GLM of rats' next choice given partner's current choice in 100% (left) and 50% (right) reward contingencies (see Materials and Methods for details). 100%: WT real versus WT shuffled: Mann–Whitney *U* test, *U* = 100.0, *p* = 0.0002; *Fmr1^−/y^* real versus *Fmr1^−/y^* shuffled: Mann–Whitney *U* test, *U* = 100.0, *p* = 0.0002; WT real versus *Fmr1^−/y^* real: *U* = 80.00, *p* = 0.0257. 50%: WT real versus WT shuffled: Mann–Whitney *U* test, *U* = 100.0, *p* = 0.0002; *Fmr1^−/y^* real versus *Fmr1^−/y^* shuffled: Mann–Whitney *U* test, *U* = 100.0, *p* = 0.0002; WT real versus *Fmr1^−/y^* real: *U* = 80.00, *p* = 0.0257. *p < 0.05, ***p < 0.001.

Rat pairs were trained to perform a cooperative learning task that required spatial coordination on W-mazes to obtain joint rewards. Each rat navigated its own W-shaped maze positioned facing its partner's maze and separated by a transparent acrylic divider that allowed visual and olfactory access with minimal physical contact (Fig. 1). Rewards were delivered only when both rats simultaneously nose-poked at spatially corresponding wells on their respective mazes. The task was continuous and self-paced, enabling extended, naturalistic interactions, and the gradual emergence of cooperative strategies over time.

Training began with a 100% reward contingency phase, in which every coordinated match, comprising simultaneous nose-pokes on corresponding maze arms, was rewarded. Once rats consistently engaged in the task and demonstrated reliable coordination, we transitioned them to a 50% contingency phase, where coordinated matches were rewarded probabilistically (*p* = 0.5). This two-phase structure was designed to first reinforce coordination through consistent reward delivery and then challenge animals to sustain cooperative behavior under reduced reward certainty—encouraging behavioral flexibility, persistence, and sensitivity to partner behavior.

Each rat pair underwent at least two training sessions per day, each lasting 20–30 min. To minimize potential maze-related biases, rats alternated between the two mazes across sessions, with assignments pseudorandomized across days.

#### Sensory-deprived control sessions (Fig. 2)

Following the establishment of proficiency in the coordination task at the 50% reward contingency, rat pairs underwent a series of control sessions designed to isolate the role of sensory cues in cooperative behavior. In these sessions, the previously used transparent barrier separating the two rats was replaced with an opaque barrier, effectively preventing the animals from seeing each other. These opaque barrier sessions were strategically interspersed among regular 50% reward sessions with full partner visibility to maintain the integrity of the learned task while allowing within-pair comparisons of cooperative performance under different sensory conditions. Additionally, to eliminate the possibility that rats could rely on auditory signals for cooperation, continuous white noise was played throughout the opaque sessions. We used lower intensity (∼65 dB) white noise that has been successfully employed as safety cues (CS−) in fear discrimination paradigms without inducing aversive reactions (Quirk et al., 1997; Tarpley et al., 2010; Takemoto and Song, 2019). This auditory masking ensured that any cooperative behavior observed was not influenced by nonvisual auditory cues, thus providing a rigorous test of the necessity of visual and auditory communication in supporting coordinated foraging between the rat pairs.

#### Visual-cue association task (Fig. 2)

To assess whether *Fmr1* rats' deficits in the cooperative learning task stem from impaired social processing or alternative sensory and cognitive impairments, we designed a visually cued reward association task. This control task evaluated each rat’s ability to detect visual stimuli, associate them with specific actions, and adapt to changing reward contingencies—skills fundamental to successful coordination in the social-W task.

Rats were trained in a W-shaped maze (80 cm × 80 cm) with three reward wells. Each well was equipped with a white LED visual cue, placed on the opposite side of a clear partition (where the partner rat would be located in the main social cooperation task). At any given time, only one well was visually cued, requiring rats to navigate and nose-poke the correct well to obtain a milk liquid reward. Upon correct responses, the LED cue remained active at the same well for ∼2 s during reward consumption, before randomly shifting to one of the two remaining wells to promote cue–reward association and facilitate learning.

Training began with a 100% reward contingency phase, where every correct response was rewarded, followed by a 50% reward contingency phase, where correct responses were rewarded with probability 0.5. Sessions were self-paced and fully automated, without a predefined trial structure. Each rat completed 1–2 daily sessions lasting 20–30 min, with performance assessed across 30 total sessions. Learning criterion was defined as achieving ≥70–80% correct responses for at least three consecutive sessions before transitioning to the 50% reward contingency phase.

As part of task shaping, rats were pretrained on a linear track to familiarize them with reward well ports and the association between ports and rewards before full task exposure.

#### Cooperation with a virtual agent (Fig. 3)

Rats were trained to navigate a W-shaped maze to obtain milk rewards that were dispensed probabilistically according to a computerized control system. This task was designed to mimic the 50% reward contingency used in our cooperative learning paradigm (Fig. 1). Specifically, the maze consisted of three arms arranged in a W configuration, each serving as a potential reward location.

On each trial, one of the three arms was designated as the rewarding arm by the computer program, with the selection made randomly to ensure unpredictability. Once a rat received a reward at a particular arm, it was required to move to either of the two remaining arms to obtain the next reward. This rule encouraged the animal to visit different arms rather than repeatedly returning to the same rewarded location.

The computer program acted as a virtual agent by determining the rewarding arm on every trial, effectively leading the sequence of trials. Rats were therefore challenged to learn and anticipate the virtual agent's random reward assignment to optimize their foraging strategy. This setup required the rats to either guess the rewarding location or cooperate implicitly with the virtual agent's programmed contingencies, enabling the study of decision-making processes under probabilistic reward conditions.

### Behavioral quantification and statistical analysis

Unless noted otherwise, behavioral analyses used the first 21 sessions for the 100% reward contingency and the first 35 sessions for the 50% reward contingency, matching the minimum available sessions across all rat pairs used across groups.

#### Performance in cooperative learning task (Fig. 1)

Behavioral performance was assessed by calculating the ratio of coordinated matches to the total number of arm transitions made by each rat pair. This measure controlled for variability in task engagement across sessions and genotypes. Performance was expressed as the percentage of matches relative to total transitions:

$$\text{\%}\;\mathrm{Performance}=\;\frac{\left[(\mathrm{Total}\;\mathrm{matches}/\mathrm{Total}\;\mathrm{transitions})\times 100\right]}{1/2}\;\;(1)$$

For an optimally performing pair, each coordinated match would require at least two transitions—one from each rat—resulting in a maximum achievable performance score of 100%. Behavioral performance was quantified as percent correct responses per session, with additional analyses of latency to correct choice and performance adaptation between 100 and 50% reward contingencies.

#### Multinomial logistic regression and cross-validation for quantifying coordination between rats (Fig. 1)

As another measure of degree of coordination between pairs of rats during the cooperative learning task on W-mazes, we implemented a multinomial logistic regression approach to evaluate how well a rat's next choice could be predicted from its partner's current location.

To assess whether a rat's location could predict its partner's subsequent choice, we trained multinomial logistic regression classifiers (using *scikit-learn* Python package) to predict the rat's next choice on its own maze based on the current position of its partner located on the other maze. The input predictor was one-hot encoded, and models were evaluated using fivefold stratified cross-validation for each rat individually. Classification accuracy was computed and averaged across folds to generate a rat-wise predictive accuracy measure.

To evaluate whether the observed accuracies were greater than expected by chance, we conducted permutation testing by independently shuffling the vectors of the focal rat's next choices and current locations of the partner rat 1,000 times for each rat and repeating the cross-validation procedure. The resulting null distribution of accuracies was used to compute empirical *p* values for each group by comparing observed mean accuracy to the distribution of permuted accuracies.

Group-wise differences in classification accuracy (both observed and null) between WT and *Fmr1* rats were evaluated using the Mann–Whitney *U* test (two-tailed).

#### Permutation testing of coordination performance (Fig. 1)

To test whether coordination improvements reflect true learning, we compared observed performance to null distributions generated by circularly time-shifting one animal's entire sequence of reward well visits within each session, thereby preserving session-level statistics while disrupting coordinated timing between animals. Each null distribution was constructed from 1,000 permutations. *p* values reflect the proportion of permutations in which surrogate performance matched or exceeded the observed value.

#### Performance quantification and statistical analysis for visual-cue association task (Fig. 2)

Behavioral performance was quantified as percent correct responses per session, with additional analyses of latency to correct choice and performance adaptation between 100 and 50% reward contingencies. A mixed-effects model was used to assess learning trajectories, incorporating random effects for individual rats and fixed effects for session, genotype, and reward contingency.

Statistical comparisons for the visual-cue association task included main effect of session (learning progression; *p* < 0.001), main effect of genotype (no significant differences; *p* = 0.981, indicating preserved visuo-cognitive performance in *Fmr1* rats), and genotype × session interaction (not significant; *p* > 0.05, suggesting similar learning trajectories between groups).

Performance data were smoothed using a Gaussian kernel (*σ* = 2 sessions) for visualization, and multiple comparisons were corrected using Bonferroni/FDR correction.

#### Performance in cooperation with a virtual agent (Fig. 3)

Behavioral performance was quantified as percent of correct rewarded responses per session.

#### Choice triplet counts, exploration efficiency, and sequence entropy (Fig. 3)

To investigate patterns of exploration and choice regularity, we analyzed the sequences of reward well visits made by individual rats during the task. Each reward well poke was recorded and used to reconstruct the animal's choice sequence over time. From these data, we defined “choice triplets” as contiguous sequences of three consecutive well visits, representing the smallest temporal unit that could capture structured exploration across multiple spatial targets.

A sliding nonoverlapping window approach was used to extract all such triplets within each behavioral session for every rat. Given the three reward wells (labeled 1, 2, and 3), eight unique choice triplets were possible: 1-2-1, 1-2-3, 1-3-1, 1-3-2, 2-1-2, 2-3-2, 3-1-3, and 3-2-3. Among these, only two triplets, 1-2-3 and 1-3-2, resulted in visits to all three wells within the span of three pokes, thereby representing maximally efficient exploration of the available reward space. These were classified as optimal choice triplets. The remaining six patterns, which revisited wells before sampling all three, were designated as suboptimal triplets as they reflect redundant or inefficient exploration paths. For each rat and each session, we quantified the frequency of occurrence of each triplet type. This allowed us to characterize how often rats explored all available reward options in a systematic manner versus exhibiting stereotyped or repetitive behaviors.

To directly quantify efficiency of exploration, we assessed how quickly and frequently each rat visited all three reward wells during a session. The rationale is that an optimally exploring rat would systematically sample the environment, rapidly visiting all wells without unnecessary repetition. In the ideal case, such a rat would encounter all three wells after just three visits, for example, via triplets like 1-2-3 or 1-3-2, and would do so repeatedly across the session.

In contrast, a rat with poor exploration efficiency might display repetitive or biased behavior, such as persistently alternating between only two wells (e.g., 1-2-1), and take more visits to cover the entire reward space. These inefficient patterns are reflected in a higher frequency of suboptimal triplets.

To operate this, we computed the proportion of optimal triplets (i.e., 1-2-3 and 1-3-2) over the total number of triplets in each session:

$$\mathrm{Exploration}\;\mathrm{efficiency}=\;\frac{\#Optimal\;choice\;triplets}{\#Total\;choice\;triplets}\;\;(2)$$

This proportion served as an index of exploratory efficiency. Higher values indicated systematic and balanced foraging across all wells, while lower values reflected redundancy or bias in choice behavior. Session-wise comparison of this metric thus provided a dynamic measure of how well the rats engaged in efficient sampling of the reward space during the coordination task.

In parallel, we also quantified the entropy of choice sequences as a separate measure of behavioral regularity. For each session, we computed the sample entropy of the full choice sequence (i.e., the sequence of visited reward wells) to capture the degree of predictability in the animal's behavior. A reduction in entropy over sessions would suggest increasingly stereotyped or regularized behavior, while persistently high entropy would reflect continued exploratory variability. Entropy values were computed for each session per rat and subsequently compared across genotypes.

#### Cross-rat choice sequence similarity analysis (Fig. S2)

To quantify behavioral choice sequence similarity between rats within a pair, we computed cross-rat Jaccard similarity matrices. For each rat pair, all sessions of the first rat were compared against all sessions of the second rat. Each matrix entry corresponded to the Jaccard similarity between the two corresponding sequences, defined as follows:

$$S_{\mathrm{Jaccard}}(\mathrm{Se}q_{\mathrm{Rat}1},\mathrm{Se}q_{\mathrm{Rat}2})=\frac{|Seq_{\mathrm{Rat}1}\cap Seq_{\mathrm{Rat}2}|}{|Seq_{\mathrm{Rat}1}\cup Seq_{\mathrm{Rat}2}|}\;\;(3)$$

where $Seq_{\mathrm{Rat}1}$ and $Seq_{\mathrm{Rat}2}$ are the sequences of maze arm transitions for a given session of each rat. Values range from 0 (no shared transitions) to 1 (identical sequences), providing a direct measure of behavioral alignment through similarity of choice sequences between paired rats.

For visualization, lower triangular versions of cross-rat similarity matrices were displayed as heatmaps, with sessions of one rat along the *x*-axis and sessions of the partner along the *y*-axis. A color gradient indicated the magnitude of similarity.

#### Cross-correlation analysis of well arrivals (Fig. 4)

To quantify temporal coordination between rats, position data were converted into discrete well occupancy by assigning each time point to the nearest well if the head position fell within a fixed radius (40 pixels) of the well center; otherwise, occupancy was set to null. Data were downsampled to 1 Hz to capture behavior at a coarse timescale. Well arrival events were defined as transitions into a well, identified as time points where the well identity differed from the preceding time point. For each rat, arrival times were converted into binary time series, where 1 indicated an arrival at a given time point and 0 otherwise. Cross-correlation between the two binary time series was computed using the correlate function from the *SciPy* signal processing library (*scipy.signal.correlate*) with “*full*” mode. The resulting cross-correlation was normalized by the product of the Euclidean norms of the two signals. Lag values were defined such that zero lag corresponded to simultaneous arrivals, positive lags indicated that Rat 2 followed Rat 1, and negative lags indicated that Rat 1 followed Rat 2. The lag corresponding to the maximum cross-correlation was taken as the dominant temporal offset between animals. Further, lag estimates were independently verified using a normalized cross-correlation approach implemented with *scipy.signal.correlate*, where correlation coefficients were computed across a range of time shifts and converted into temporal delays based on the sampling rate. To compare coordination across experimental groups (e.g., WT vs *Fmr1*), distributions of peak cross-correlation lags were aggregated across sessions and compared using the two-sample Kolmogorov–Smirnov (KS) test (*scipy.stats.ks_2samp*). In addition, kernel density estimates (KDEs) of the lag distributions were computed for visualization using the Seaborn library (*seaborn.kdeplot*). In addition to full-session analyses, cross-correlations were also computed within temporal windows centered on individual arrival events to assess local coordination dynamics.

#### Assignment of leader and follower identities for matched events (Fig. 5)

For each matched event, the rat that arrived first at the reward well on its respective W-maze arm was designated as the Leader, while the other rat was assigned as the Follower. These roles were determined based on the precise timing of their reward well entries (poke timings).

#### State-space modeling of leadership probability (Fig. 5)

To estimate the dynamic probability of a rat leading during each matched event across sessions, we applied a state-space modeling approach to the binary leadership vector derived for each rat. The vector encoded leadership status per event, with 1 indicating the rat led the event and 0 indicating it followed.

Following the framework described previously (Smith et al., 2004), we modeled the latent, time-varying propensity to lead as a hidden continuous state that evolves according to a Gaussian random walk. Observed binary leadership outcomes were assumed to arise from a Bernoulli process with a logistic link function mapping the latent state to a probability of leading.

Parameter inference was performed using an expectation-maximization (EM) algorithm combined with a Newton–Raphson procedure to update latent states. This algorithm iteratively estimated the smoothed latent state trajectory, the associated posterior variance, and the time-varying probability of leading, while updating process noise parameters until convergence.

Confidence intervals for the latent state and the resulting probability estimates were calculated using numerically integrated posterior distributions. The model was implemented with performance optimizations including Just-In-Time compilation (*Numba*) and batch processing of confidence limits.

In brief, this approach allowed for the quantification of uncertainty in leadership probability over time, yielding smooth, probabilistic estimates of leadership dynamics across matched events within and between sessions.

We implemented a state-space model (Smith et al., 2004) to estimate the latent, time-varying probability of leadership from binary observations of leadership per matched event.

The hidden state *x_k_* evolves as a Gaussian random walk:

$$x_{k}=x_{k\text{-}1}+\epsilon_{k},\;\epsilon_{k}\;∼\;N(0,\;v_{e})\;\;(4)$$

where *v*_e_ is the process noise variance.

The observed binary leadership outcome *n_k_* ∈ {0,1} at time *k* is modeled as a Bernoulli trial with probability:

$$p_{k}=Pr(x_{k})=\;\frac{1}{1+e^{-(b_{0}+\;x_{k})}}\;(5)$$

The goal is to estimate the posterior distribution of the latent states {*x*_k_} and process noise *v*_e_ given the observations {*n*_k_}. Due to the nonlinear logistic observation model, exact inference is intractable.

So, we used an Expectation-Maximization (EM) algorithm combined with a Newton–Raphson update at each time step to iteratively estimate the latent states and optimize *v*_e_. The Newton–Raphson step solves:

$$x_{k}^{\left(i+1\right)}=\;x_{k}^{\left(i\right)}-\;\frac{\;f(x_{k}^{\left(i\right)})}{{\;f}^{\prime }(x_{k}^{\left(i\right)})}\;\;(6)$$

where

$$f(x_{k})=x_{k}-\;x_{k}^{\mathrm{pred}}-\;v_{k}^{\mathrm{pred}}\left(n_{k}-\frac{e^{b_{0}+\;x_{k}}}{1+e^{b_{0}+\;x_{k}}}\right)\;\;(7)$$

and

$$f^{\prime }(x_{k})=\;1+\;v_{k}^{\mathrm{pred}}\frac{e^{b_{0}+\;x_{k}}}{{\left(1+e^{b_{0}+\;x_{k}}\right)}^{2}}\;\;(8)$$

Here, $x_{k}^{\mathrm{pred}}$ and $v_{k}^{\mathrm{pred}}$ are the predicted state mean and variance from the previous iteration.

The model yields smoothed estimates of the latent state *x*_k_, from which the time-varying leadership probability *p*_k_ and confidence intervals are computed.

#### Multiagent reinforcement learning framework for modeling cooperative behavior (Fig. 6)

To complement behavioral findings and test whether access to partner information facilitates cooperative behavior, we developed a multiagent reinforcement learning (RL) model of the spatial coordination task (Tan, 1993; Du et al., 2023; Tsutsui et al., 2024). In this model, two agents independently navigated discrete three-location grids and were required to coordinate their choices to receive a shared reward. We implemented three agent types with varying access to task-relevant cues: partner-aware agents received both self- and partner-location information; partner-unaware agents received only self-related location information; and random agents received no observations and selected actions uniformly at random. This design allowed us to isolate the role of partner information in driving coordination over the course of learning.

The environment consisted of two agents, each occupying its own discrete 1D grid composed of three locations, representing the reward wells in the behavioral task. On each timestep, one agent was randomly selected to act first. This agent observed the current environmental state—including its current and previous locations, the location of the previously rewarded match, and, for partner-aware agents, the partner's current location—and then selected an action. The second agent subsequently observed the updated environment state, which included the same set of features along with the first agent's new position and selected its action accordingly. A joint reward of +1 was delivered only if both agents selected the same location and that location differed from the previously rewarded location. Random agents received no observations and chose actions randomly. This framework preserved the task's turn-taking dynamics and nonrepetition rule while allowing precise manipulation of partner cue access.

Partner-aware and partner-unaware agents learned using standard tabular Q-learning. For each agent, a state–action value table was maintained and updated based on the following rule:

$$Q(s,a)\leftarrow Q(s,a)+\alpha [r+\gamma \;*\;\mathrm{ma}x_{a^{\prime }}\;Q(s^{\prime },\;a^{\prime })-Q(s,a)]\;\;\;(9)$$

where *Q*(*s*, *a*) is the estimated value of taking action *a* in state *s*, *α* is the learning rate, *γ* is the discount factor, *r* is the reward received, and *s*ʹ is the resulting state after the action. Agents selected actions using an epsilon-greedy policy to balance exploration and exploitation. The probability of random exploration (epsilon) was decayed exponentially across episodes to encourage early exploration and later convergence on learned policies. Specifically, epsilon decayed from 1.0 to 0.01 over 90% of the training episodes according to the following:

$$\epsilon_{t}=\epsilon_{0}\;*\;e^{\left(-\lambda t\right)}\;\;(10)$$

where $\epsilon_{0}$ is the initial exploration rate and λ is the decay rate calculated to ensure that $\epsilon_{t}=\;0.01$ by the end of the decay window. After this point, epsilon was held constant at 0.01 for the remainder of training.

#### Position tracking for gaze analysis (Fig. 7)

DeepLabCut 2.3.9 (Mathis et al., 2018) was utilized to track the position of different body parts in videos of rats performing the behavioral task. Prior to model training, each video recording was processed to generate two separate datasets, corresponding to the rat located on the left W-maze and the rat located on the right W-maze. This involved cropping the original videos to isolate and extract the respective rat's movements within each W-maze. Then, 20–40 frames from at least 10 videos (from each experimental cohort) were automatically extracted (using *k-means algorithm*) for labeling the keypoints and training the model, thus capturing the variability in posture and luminance conditions. The extracted frames were manually labeled to define the keypoints corresponding to different body parts. A ResNet-50 based model was trained with 95% of the labeled data with default parameters for 100,000 number of training iterations. Keypoints with likelihood <0.6 were set to NaN, interpolated, and the resulting positions smoothed with a Gaussian kernel (*σ* = 150 ms). We then used a *p*-cutoff of 0.6 to condition the *x*- and *y*-coordinates for future analysis. This network was then used to analyze videos from similar experimental settings.

#### Quantification of peer-directed head orientation events (Fig. 7)

To quantify social attention, we analyzed partner-directed head orientation using keypoint tracking data from DeepLabCut. Analysis was restricted to time periods meeting two criteria: (1) the rats were positioned at distinct, nonopposing reward wells on their respective W-mazes, ensuring spatial separation; and (2) the focal rat was classified as the Follower if it transitioned to a new reward well while the partner remained stationary (waiting at its reward well), thereby making a cooperative or noncooperative choice. A trial was defined as the interval beginning when the rats were in the initial configuration at reward wells and ending when the Follower departed its well to transition to the next well. For each trial, we quantified partner-directed head orientation from the Follower to the Leader, from the Leader to the Follower, and mutually oriented head orientation.

We calculated a head direction vector (*v*^head^) for the focal rat, defined from the neck to the head center, and a partner vector (*v*^partner^), defined from the focal rat's head center to the partner's body center.

The bearing angle θ was computed using the following:

$$\theta =\mathrm{co}s^{-1}\left(\frac{v^{\mathrm{head}}.v^{\mathrm{partner}}}{\left|\left|v^{\mathrm{head}}\right||.|\left|v^{\mathrm{partner}}\right|\right|}\right)\;(11)$$

A head orientation event was classified as peer-directed if θ fell below a predefined angular threshold, indicating that the focal rat was directing its head toward the partner. We adopted an angular threshold of 40°, in line with criteria used by Wallace et al. (2013). Supplementary analyses with different thresholds yielded qualitatively similar results, indicating that our findings were not overly sensitive to the specific threshold choice.

We then extracted three key summary metrics: (1) the proportion of time the Follower rat oriented toward the Leader (*g*_F_) and (2) the proportion of time both rats simultaneously oriented toward each other (*g*_M_). These metrics quantified the prevalence and mutuality of social attention between the dyad members.

#### Model structure for gaze analysis (Fig. 7)

The mixed-effects logistic regression model included: all main effects for gaze measures, genotype, and session blocks, all two-way and three-way interactions between predictors, random intercepts for individual animals, random intercepts for sessions nested within animals.

The model was specified as follows:

$$\mathrm{logit}(P(\mathrm{match}))\;∼\;(g_{F}+g_{M})\;*\;\mathrm{Genotype}\;*\;\mathrm{Session}\;\mathrm{Block}+(1\;|\mathrm{animal}/\mathrm{Session})\;\;(12)$$

where:

*g*_F_ and *g*_M_ denote normalized follower-only and mutual gaze durations, respectively.

Genotype denote the genotype of the rat.

Session Block denotes the session block (early, mid, late) nested within animal.

#### Statistical implementation for gaze analysis (Fig. 7)

All analyses were conducted in R (version 4.4.3). The mixed-effects model was fitted using the function from the *glmmTNB* package with the binomial family and logit link function. Model convergence was verified.

#### Post hoc analyses for gaze analysis (Fig. 7)

To interpret significant interactions, we conducted post hoc contrast analyses using the *emmeans* package. Specifically:

Within-genotype contrasts: We computed estimated marginal trends (*emtrends*) for each gaze measure across session blocks within each genotype, followed by pairwise contrasts to test for significant changes across training phases.

Between-genotype contrasts: We compared estimated marginal trends between genotypes within each session block to identify when genotype differences emerged.

All post hoc comparisons were adjusted for multiple testing using the Tukey method. Statistical significance was set at *α* = 0.05 for all tests.

#### Model evaluation for gaze analysis (Fig. 7)

Model fit was assessed using marginal *R*^2^ (variance explained by fixed effects only) and conditional *R*^2^ (variance explained by the complete model including random effects). Results are reported as odds ratios with 95% confidence intervals and *p* values.

#### Partner behavior history analysis (Fig. 8)

To test whether rats adjust their cooperative decisions based on recent partner behavior—and whether this differs by genotype—we analyzed trial-by-trial choices during “follower trials.” In each trial, one rat (the leader) enters an arm first; the other rat (the follower) then decides whether to choose the matching arm and cooperate. Leader and follower roles alternate dynamically within each session, allowing both rats to serve in both roles.

For each follower trial, we aligned the partner’s most recent choice (from their previous follower trial) and computed the elapsed time between that choice and the current follower decision (Δ*t*) for use in the statistical model. Trial sequences for each rat were constructed by sorting all trials chronologically and identifying those where the rat served as Follower. Partner history variables were derived by matching each follower trial to the most recent follower trial completed by the partner.

#### Generalized linear mixed-effects modeling of partner influence (Fig. 8)

To quantify how recent partner behavior influenced cooperative decisions, and how this relationship differed by genotype, we fit a generalized linear mixed-effects model (GLMM) predicting the likelihood of cooperation on each follower trial (binary outcome: cooperate = 1, not cooperate = 0).

Fixed effects included the partner’s previous choice (binary), the elapsed time since that choice (Δ*t*, continuous), and their interaction. Genotype was modeled as an interaction term with all predictors to assess genotype-specific effects.

We also included covariates reflecting the subject’s own recent choice history to control for individual behavioral tendencies. Δ*t* was included to account for intertrial timing differences, particularly the longer partner-to-subject intervals observed in *Fmr1* rats.

Random intercepts for rats and sessions accounted for repeated measures. Separate models were fit for the 100% and 50% reward contingencies to isolate effects within each condition.

Models were implemented in Python using the *pymer4* package (v0.8) and validated against equivalent R-based implementations (*lme4*).

### Statistical analysis

All statistical analyses were conducted using Python (*scipy*, *statsmodels*) or RStudio (*lme4*, *afex*, *effects*, *emmeans*, *glmmTNB*).

### Data and code availability

All data underlying these results will be uploaded in the NWB (Neurodata Without Borders) format to DANDI (ID: 001845) upon acceptance. Code to replicate these results will be available on our lab GitHub (https://github.com/JadhavLab/Social_CoOp_Behavior) upon acceptance.

## Results

### A spatial cooperation task for social coordination in rat pairs

We developed a spatial cooperation task in which littermate rat pairs (dyads) from a *Fmr1* knock-out (*Fmr1)* rat colony must simultaneously choose matching reward wells from three options on complementary W-mazes separated by transparent barriers. Each rat navigates its own maze but must learn to coordinate actions with its partner on the other maze to visit matching reward locations on complementary arms of the two mazes and simultaneously nose-poke in the reward wells to receive automatically dispensed rewards (Fig. 1*A*,*B*; see Materials and Methods).

In a behavioral shaping step, rat pairs were first trained on parallel linear tracks separated by a transparent, perforated barrier and rewarded for simultaneous nose-pokes in matching reward wells, facilitating the transition from individual to coordinated actions (Fig. 1*C*). Rat pairs were then introduced to the spatial cooperative task on paired W-mazes, first with a deterministic 100% reward contingency for each “match”/coordinated event of nose-poking on matched reward wells, resulting in both rats receiving rewards in their respective wells for every successful match event. After training on several sessions with 100% rewards over multiple days, the task was then switched to a 50% probabilistic reward contingency, in which the rats were only rewarded with a random 50% chance for each successful match event (Fig. 1*C*; examples of successful and unsuccessful cooperation shown in Movie 1).

**Movie 1.** Video showing examples of successful and unsuccessful cooperation events between two rats. The first example trial illustrates effective coordination between two rats resulting into successful cooperation on the task. The second example trial illustrates failed coordination between two rats resulting into unsuccessful cooperation. [View online]

Rats performed at least two behavioral sessions per day interleaved with a rest/sleep epoch, and each individual rat is run on both mazes within a day (switching assigned maze after each session). To assess behavioral flexibility and promote optimal exploration of all three maze arms, we trained rat pairs on the 50% probabilistic reward contingency. For the deterministic 100% contingency, each match event leads to reward, so rat pairs can choose to simply run back and forth between any two reward well arms of their maze in a coordinated manner, similar to the linear maze. In contrast, in the 50% probabilistic condition, after each successful rewarded “match” event on a given maze arm, only one of the remaining two arms was randomly chosen as the reward-arm for the next transition. Both animals therefore must together explore the remaining two possible options until they get rewarded, encouraging optimal exploration of all three arms of the mazes. There is 50% probability of match events being rewarded, and animals must continue coordinating actions (spatial trajectories), even if they do not get rewarded after a match event, requiring cooperative behavior irrespective of reward success. Coordination of well-visits on opposing arms therefore becomes a primary goal of the task, which must be learnt together by rat pairs.

Adult male littermate rat pairs (dyads)—WT-WT (WT pairs), *Fmr1-Fmr1* (*Fmr1* pairs), and WT-*Fmr1* (mixed pairs), with *N* = 5 pairs per group—were trained on this cooperative task. Cooperative behavior learning and performance over sessions for an example WT and *Fmr1* pair for both the 100 and 50% contingencies are shown in Figure 1*D*. Both WT and *Fmr1* rat pairs exhibited performance indistinguishable from chance during early training on both reward contingency tasks; however, by late training, their performance improved significantly, exceeding chance levels (Fig. 1*H*,*I*). Importantly, WT pairs demonstrated significantly higher coordination rates than both *Fmr1* pairs (Fig. 1*E*,*I*) and mixed pairs (Fig. S1*A*) under both 100% and 50% reward contingencies. *Fmr1* and mixed pairs were generally slower in the task, as reflected by a reduced number of task-related transitions (Fig. 1*F*,*G*; Fig. S1*D*), but even accounting for this difference, their learning and performance in cooperative behavior was impaired (Fig. 1*I*, Fig. S1*B*). Consistent with enhanced learning and performance, the current location of a peer provided sufficient information to reliably predict a rat's subsequent response (Fig. 1*J*, Fig. S1*C*). This predictive relationship exceeded chance levels across all rat pairs. However, prediction accuracy was significantly higher in WT pairs compared with both *Fmr1* and mixed-genotype pairs, highlighting a genotype-dependent modulation of social predictive cues. Taken together, these results demonstrate that while all rat pairs were capable of learning to coordinate, WT pairs exhibited significantly higher cooperative performance, faster transitions in trajectories between reward wells, and enhanced sensitivity to peer cues. These findings suggest that *Fmr1* rats exhibit specific impairments in the acquisition and use of social information critical for effective cooperation.

### Cooperation deficits in *Fmr1* rats are not due to sensory or learning deficits

**Figure 2.**
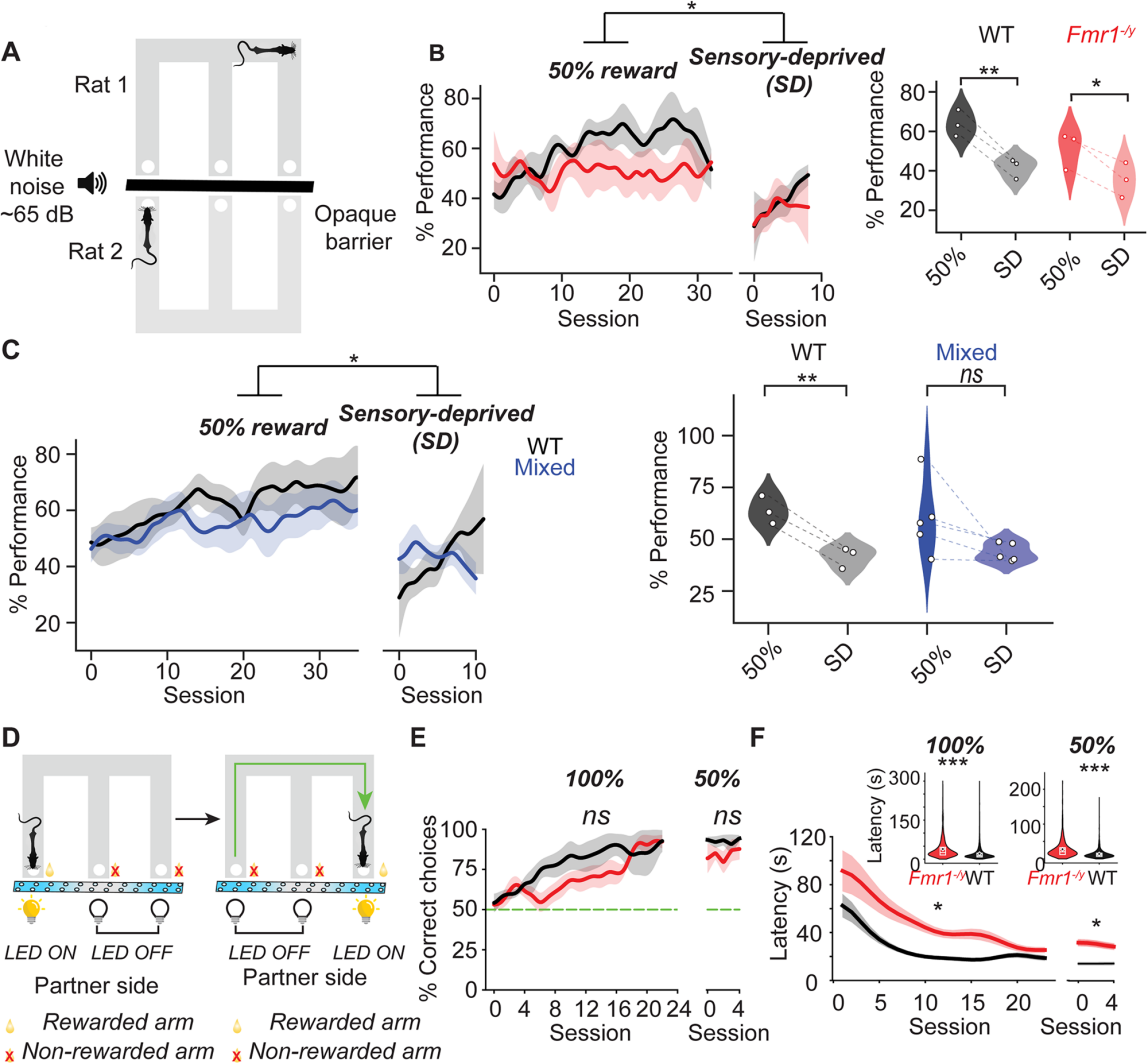
Behavioral controls. ***A***, Schematic showing sensory-deprived (SD) control sessions. Rats in a pair ran on their respective mazes with an opaque barrier between mazes obscuring sight. ***B***, Left: Session-wise comparison of performance of rat pairs between 50% reward (WT: *N* = 3 pairs; *Fmr1^−/y^*: *N* = 3 pairs) and sensory-deprived conditions. Two-way RM ANOVA (genotype: *F*_(1,4)_ = 1.5358, *p* = 0.28299, condition: *F*_(1,4)_ = 9.5069, *p* = 0.03681, genotype × condition: *F*_(1,4)_ = 0.0181, *p* = 0.89953). Right: Comparison of mean performance of rat pairs between transparent (last 9 sessions, *N* = 3 pairs) and opaque conditions (*N* = 3 pairs). Paired *t* test (WT: *t* = 12.074, *p* = 0.0068, *Fmr1^−/y^*: *t* = 5.227, *p* = 0.0347).*p < 0.05, **p < 0.01. ***C***, Left: Session-wise comparison of performance of rat pairs between 50% reward (WT: *N* = 3 pairs; Mixed: *N* = 5 pairs) and sensory-deprived conditions. Two-way RM ANOVA (genotype: *F*_(1,6)_ = 0.0343, *p* = 0.085910, condition: *F*_(1,6)_ = 7.8071, *p* = 0.03141, genotype × condition: *F*_(1,6)_ = 0.06, *p* = 0.80880). Right: Comparison of mean performance of rat pairs between standard and sensory-deprived conditions. Paired *t* test (WT: *t* = 12.074, *p* = 0.0068, Mixed: *t* = 2.050, *p* = 0.0625). ns, not significant, **p < 0.01. ***D***, Schematic for visual cue association task. Rats were trained on a W-maze to approach an LED-cued arm on the opposite maze to seek milk rewards on the corresponding, apposing reward well on it’s own maze. ***E***, Performance comparison between WT and *Fmr1^−/y^* rats on the visual cue-association task under 100% (left) and 50% (right) reward contingencies (WT: *N* = 4 rats; *Fmr1^−/y^*: *N* = 4 rats). Lines represent the smoothed mean across animals; shading indicates SEM; the dashed line denotes chance performance. 100%: Linear mixed effects model (genotype: *F*_(1,7.7)_ = 0.825, *p* = 0.391, session: *F*_(1,152.68)_ = 233.229, *p* = 1.50 × 10^−32^, genotype × session: *F*_(1,152.68)_ = 1.524, *p* = 0.219). 50%: Linear mixed effects model (genotype: *F*_(1,32.31)_ = 1.661, *p* = 0.207, session: *F*_(1,34.32)_ = 1.101, *p* = 0.301, genotype × session: *F*_(1,34.32)_ = 1.045, *p* = 0.314). ns: not significant. ***F***, Top: WT rats respond to the visual LED cue with lower latency relative to *Fmr1^−/y^* rats under both 100% (left) and 50% (right) reward contingencies. 100%: *Fmr1^−/y^*: *n* *=* 2,371, mean = 42.2 s; WT: *n* *=* 4,554, mean = 22.0 s; *p* = 6.9 × 10^−^^227^, Mann–Whitney *U* test). 50%: *Fmr1^−/y^*: *n* = 862, mean = 28.4 s; WT: *n* *=* *1*,359, mean = 14.0 s; *p* = 1.3 × 10^−^^82^, Mann–Whitney *U* test. Bottom: Latency to respond to visual cue for sessions under 100% (left) and 50% (right) reward contingencies. 100%: Linear mixed effects model (genotype: *F*_(1,6.18)_ = 5.386, *p* = 0.058, session: *F*_(1,6816.19)_ = 763.934, *p* = 1.79 × 10^−159^, genotype × session: *F*_(1,6816.19)_ = 25.391, *p* = 4.80 × 10^−7^). 50%: Mixed effects model (genotype: *F*_(1,6.18)_ = 5.050, *p* = 0.064, session: *F*_(1,2198.07)_ = 13.454, *p* = 2.54 × 10^−4^, genotype × session: *F*_(1,2198.07)_ = 1.743, *p* = 0.187). All mixed-effects models included genotype and session as fixed effects and animal as a random intercept.

To further isolate the factors that contribute to successful coordination in WT rats, and the impairment of such coordination in *Fmr1* rats, we conducted a series of targeted control experiments. First, to determine the contribution of social vision, we replaced the transparent, perforated divider with an opaque barrier (sensory-deprived condition/SD) that prevented exchange of visual information between rats (Fig. 2*A*), testing whether successful cooperation could be achieved through either random transitions between maze arms or through use of nonvisual strategies such as rhythmic coordinated transitions between maze arms. These sessions were interspersed with the standard 50% reward condition sessions, during which the rats could see each other. Compared with the 50% reward condition, rats showed a significant reduction in performance in the SD sessions (Fig. 2*B*,*C*), demonstrating that visual access to the partner, and possibly peer-directed attention, is necessary for successful joint coordination. Importantly, all experiments were conducted in the presence of continuous auditory white noise to mask potential auditory communication, thereby ruling out ultrasonic vocalizations as a primary modality for coordination.

Second, to assess whether the lower cooperation performance could be due to differences in associative learning (associating partner presence with reward), since memory deficits have been reported in *Fmr1* rats (Asiminas et al., 2019; Fernandes et al., 2021), we tested whether WT and *Fmr1* rats could form similar associations in a non-social context by replacing the social partner with a localized visual cue presented at one of the reward wells (Fig. 2*D*). As in the main task, this control was conducted under both 100% and 50% reward contingencies. Both WT and *Fmr1* rats learnt to approach the reward-associated arm based on the visual cue (Fig. 2*E*), demonstrating intact visual cue-reward associative learning. *Fmr1* rats did exhibit slower learning rates (Fig. 2*E*) and delayed approach behavior compared with WT rats (Fig. 2*F*), reflecting differences in task performance speed, rather than deficits in learning or sensory processing. Together, these results underscore the crucial role of visual cues in supporting successful coordination in WT, *Fmr1*, and mixed pairs, while demonstrating that *Fmr1* rats retain visual processing and visual-reward association capabilities, indicating that deficits in these factors cannot solely account for their impaired performance in the cooperative foraging task.

### Development of structured spatial and temporal coordination strategies supports cooperative behavior

We next examined whether rats developed structured strategies that supported efficient joint exploration, a critical component of coordinated behavior. Beyond moment-to-moment monitoring and reactive actions based on partner location, successful cooperation may require higher-order planning across extended sequences. To address this, we analyzed how pairs coordinated both the spatial structure of their exploration and the temporal alignment of their actions.

**Figure 3.**
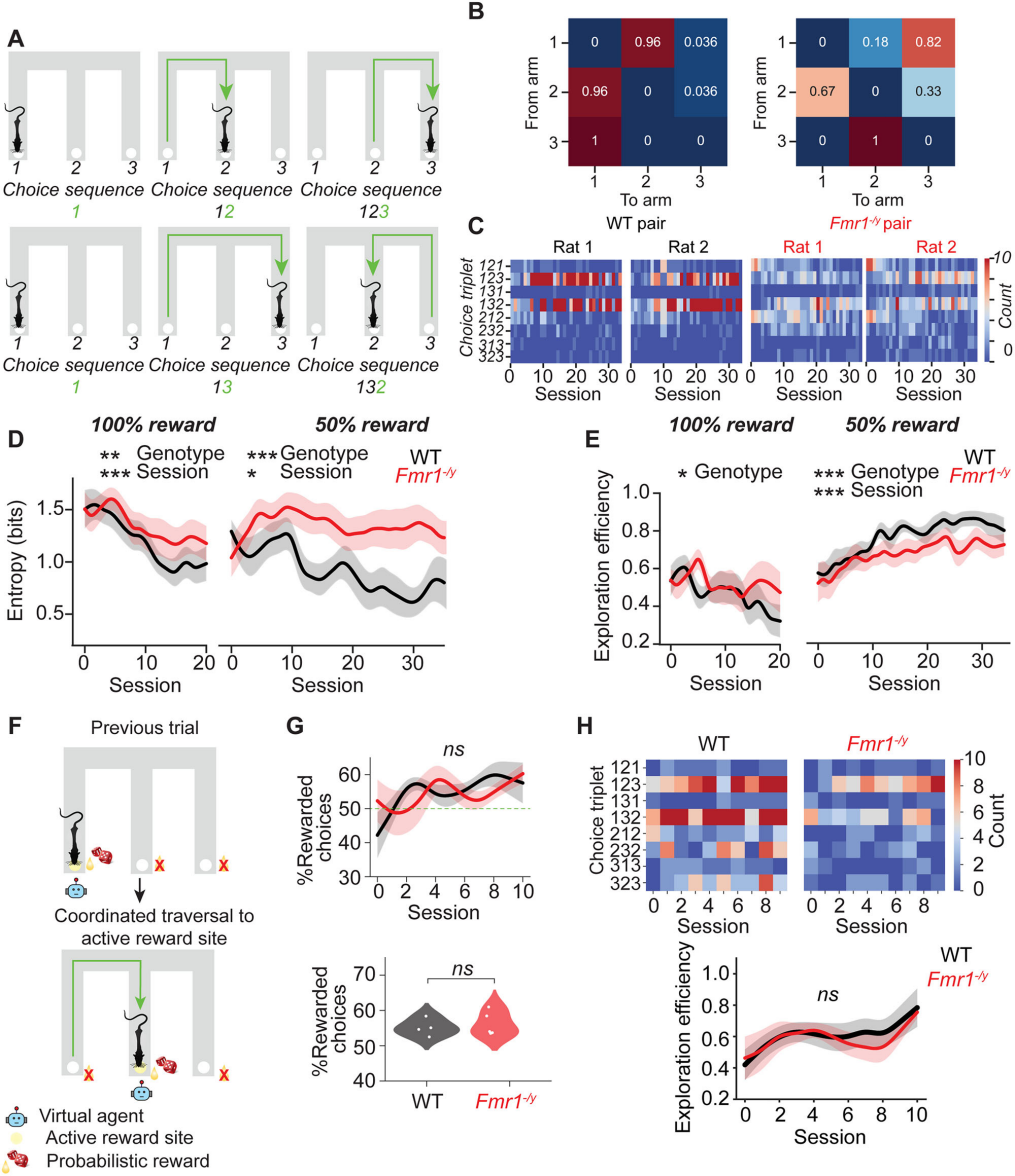
Emergence of optimal spatial coordination strategies. ***A***, Two example spatial trajectory choice sequences (top and bottom) that lead to optimal sampling of available reward space. ***B***, Well-to-well transition probabilities from an example WT session under 100% (left) and 50% (right) reward contingencies. Note the change in transition probabilities across the two reward contingencies. ***C***, Counts of unique choice triplets in 50% reward contingency sessions for rats from a representative WT pair and a representative *Fmr1^−/y^* pair. ***D***, Session-wise comparison of entropy of choice sequences of WT and *Fmr1^−/y^* rats during 100% (left) and 50% (right) reward contingencies, showing significantly higher decrease in entropy with learning for WT rats. *N* = 10 rats each for WT and *Fmr1^−/y^*. 100%: Two-way RM ANOVA (genotype: *F*_(1,378)_ = 9.0872 *p* = 0.002748, session: *F*_(20,378)_ = 4.4551, *p* = 1.888 × 10^−9^, genotype × session: *F*_(20,378)_, *p* = 0.565329). 50%: Two-way RM ANOVA (genotype: *F*_(1,630)_ = 112.9572, *p* < 2 × 10^−16^, session: *F*_(34,630)_ = 1.6380, *p* = 0.01365, genotype × session: *F*_(34,630)_ = 1.2891, *p* = 0.12879). **p < 0.01, ***p < 0.001. ***E***, Exploration efficiencies of WT and *Fmr1^−/y^* rats during 100% (left) and 50% (right) contingency sessions, showing significantly higher exploration efficiency for WT rats in the 50% condition. 100%: Two-way RM ANOVA (genotype: *F*_(1,378)_ = 4.0001, *p* = 0.04621, session: *F*_(20,378)_ = 0.9159, *p* = 0.56701, genotype × session: *F*_(20,378)_ = 0.7830, *p* = 0.73470). 50%: Two-way RM ANOVA (genotype: *F*_(1,594)_ = 42.6457, *p* = 1.410 × 10^−10^, session: *F*_(32,594)_ = 3.9482, *p* = 1.116 × 10^−11^, genotype × session: *F*_(32,594)_ = 0.8307, *p* = 0.7339). *p < 0.05, ***p < 0.001. ***F***, Schematic for agent simulation experiments. Rats learned to transition between maze arms to receive rewards with reward contingency predetermined by a computer program, similar to the 50% reward contingency in the cooperative foraging task. ***G***, Top: Session wise performance of WT and *Fmr1^−/y^* rats in the agent simulation experiments were similar to each other. *N* = 4, 5 rats for WT and *Fmr1^−/y^* respectively. Two-way RM ANOVA (genotype: *F*_(1,56)_ = 0.0032, *p* = 0.9549, session: *F*_(7,56)_ = 1.0747, *p* = 0.3918, genotype × session: *F*_(7,56)_ = 1.4660, *p* = 0.1983). Bottom: Mean performance was same across genotypes (Mann–Whitney *U* test, *p* > 0.05). ns, not significant. ***H***, Top: Heatmap of unique choice triplet counts for an example WT (left) and *Fmr1^−/y^* (right) rat. Bottom: Exploration efficiency of WT and *Fmr1^−/y^* rats were similar. Two-way RM ANOVA, genotype (*F*_(1,56)_ = 0.0771, *p* = 0.7823, session: *F*_(7,56)_ = 1.4762, *p* = 0.1946, genotype × session: *F*_(7,56)_ = 0.4601, *p* = 0.8591). ns*,* not significant.

We first compared exploration patterns across reward contingencies with differing task demands. Under the 100% reward condition, coordinated alternation between any two arms was sufficient to maximize reward, whereas the 50% probabilistic condition required systematic sampling of all three arms for optimal exploitation. Reward was maximized when rats alternated through one of two optimal arm sequences (maze arm transitions 1-2-3 or 1-3-2; Fig. 3*A*). In line with these differing requirements, transition probabilities between maze arms showed distinct patterns across contingencies (Fig. 3*B*). Parsing each session’s transitions into nonoverlapping triplets revealed that rats in the 100% condition predominantly alternated between two adjacent arms, while those in the 50% condition engaged in comprehensive exploration of all three arms through either the 1-2-3 or 1-3-2 sequences, especially for WT pairs (Fig. 3*B*,*C*). Crucially, WT pairs exhibited greater coordination in executing optimal triplet sequences with partners than *Fmr1* pairs (Fig. 3*C*,*E*). WT rats also flexibly alternated between the two optimal sequences within and across sessions and adapted strategies according to task demands (Fig. 3*C*). Further, the entropy of their choice sequences declined over training, reflecting increasing regularity and efficiency (Fig. 3*D*).

Building on the finding that WT rats developed more structured and efficient individual exploration than their *Fmr1* counterparts, we next assessed coordination between partners within rat pairs. Under the 100% reward condition, Jaccard similarity (Fig. S2*A*; see Materials and Methods) between the choice sequences of paired rats was high for both genotypes (Fig. S2*B,C*). In contrast, under the 50% probabilistic condition, WT pairs exhibited significantly higher similarity in their choice sequences compared with *Fmr1* pairs (Fig. 3-1*B,C*), indicating that only WT rats converged on shared, structured strategies when task demands were elevated. Importantly, during the sensory-deprived condition, where direct partner monitoring was eliminated, similarity decreased comparably across genotypes, suggesting that the enhanced coordination observed under normal social conditions was specifically driven by partner-based interactions (Fig. S2*D*).

To test whether these differences reflect coordination per se rather than baseline differences in exploration efficiency or cognitive ability, we introduced a control task in which rats coordinated with a virtual agent under a 50% reward contingency (Fig. 3*F*). In this task, both WT and *Fmr1* rats achieved similar levels of task performance with comparable learning rates (Fig. 3*G*). As in the 50% reward condition of the original social task, optimal outcomes required systematic alternation through the maze arms using either of the two triplet sequences (1-2-3 or 1-3-2). Notably, both genotypes independently demonstrated the capacity to adopt such structured transition strategies when interacting with the virtual agent (Fig. 3*H*), indicating that efficient and temporally extended exploration strategies were not impaired in *Fmr1* rats when peer coordination demands were not required. This suggests that WT rats exhibit flexibility in adapting their exploration strategy, likely through an internal model of partner strategy that can be updated as required using peer-monitoring, a process that is impaired in *Fmr1* rats.

**Figure 4.**
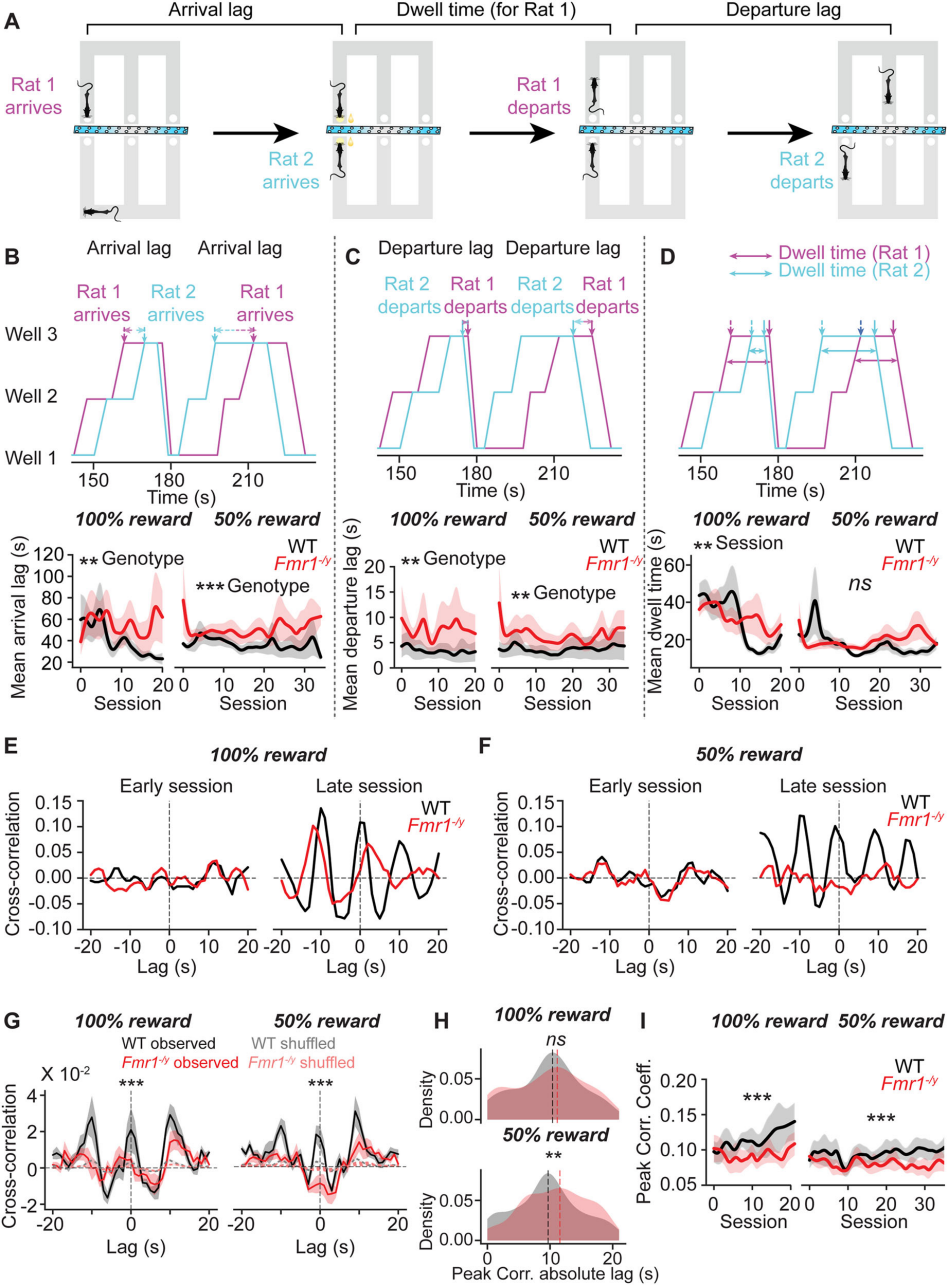
Emergence of temporal coordination and timescales of behavioral organization. ***A***, Schematic showing a sequence of events between rats in a pair. Specifically, three different events were identified: arrival lags, departure lags, and dwell time (see Materials and Methods for details). ***B***, Top: Left: Snippet showing arrival lag between rats for a cooperative event. Bottom: Left: Mean arrival lag between rats in WT and *Fmr1^−/y^* pairs for 100% (left) and 50% reward contingencies (right). *N* = 5 pairs in each group; 100%: Two-way RM ANOVA (genotype: *F*_(1,168)_ = 7.6763, *p* = 0.006225, session: *F*_(20,168)_ = 0.9020, *p* = 0.585036, genotype × session: *F*_(20,168)_ = 0.9342, *p* = 0.544923). 50%: Two-way RM ANOVA (genotype: *F*_(1,280)_ = 28.1429, *Fmr1^−/y^ p* = 2.293 × 10^−7^, session: *F*_(34,280)_ = 0.5140, *p* = 0.9984, genotype × session: *F*_(34,280)_ = 0.4752, *p* = 0.9947). ***p < 0.001. ***C***, Top: Middle: Snippet showing departure lag between rats after a cooperative event. Bottom: Middle: Mean departure lag between rats in WT and *Fmr1^−/y^* pairs for 100% (left) and 50% reward contingencies (right). *N* = 5 pairs each; 100%: Two-way RM ANOVA (genotype: *F*_(1,168)_ = 17.0918, *p* = 5.612 × 10^−5^, session: *F*_(20,168)_ = 0.2244, *p* = 0.9998, genotype × session: *F*_(20,168)_ = 0.2383, *p* = 0.9997). 50%: Two-way RM ANOVA (genotype: *F*_(1,264)_ = 21.1217, *p* = 6.686 × 10^−6^, session: *F*_(32,264)_ = 0.4528, *p* = 0.9957, genotype × session: *F*_(32,264)_ = 0.3564, *p* = 0.9996). ***p < 0.001. ***D***, Top: Right: Snippet showing dwell time at a reward well for rats’ every visit. Bottom: Right: Mean dwell time at reward wells for WT and *Fmr1^−/y^* rats for 100% (left) and 50% reward contingencies (right). *N* = 10 rats in each group; 100%: Two-way RM ANOVA (genotype: *F*_(1,378)_ = 1.3544, *p* = 0.2451, session: *F*_(20,378)_ = 3.0281, *p* = 1.731 × 10^−5^, genotype × session: *F*_(30,378)_ = 1.38, *p* = 0.1267). 50%: Two-way RM ANOVA (genotype: *F*_(1,630)_ = 3.4673, *p* = 0.06306, session: *F*_(34,630)_ = 1.1138, *p* = 0.30378, genotype × session: *F*_(34,378)_ = 1.3229, *p* = 0.10665). ns, not significant, **p < 0.001. ***E***, Example cross-correlation (smoothened) between well arrival times of an example WT and *Fmr1^−/y^* rat pair for a representative early (left) and late session (right) under 100% reward condition. ***F***, Example cross-correlation between well arrival times of an example WT and *Fmr1^−/y^* rat pair for a representative early (left) and late session (right) under 50% reward condition. Note the lack of systematic cross-correlation peaks for *Fmr1^−/y^* rat pairs in the 50% condition even after training. ***G***, Cross-correlation coefficient values between well arrival times across 100% (left) and 50% (right) reward conditions for WT and *Fmr1^−/y^* pairs. These values were quantified for each pair session-wise and averaged across pairs and sessions for both observed and shuffled data. 100%: Linear mixed-effects model (genotype: *F*_(1,8524)_ = 18.3661, *p* = 1.843 × 10^−5^, lag: *F*_(40,8524)_ = 6.9941, *p* < 2.2 × 10^−16^, genotype × lag: *F*_(40,8524)_ = 2.9710, *p* = 1.147 × 10^−9^). 50%: Linear mixed-effects model (genotype: *F*_(1,14094)_ = 94.3281, *p* < 2.2 × 10^−16^, lag: *F*_(40,14094)_ = 18.4252, *p* < 2.2 × 10^−16^, genotype × lag: *F*_4(0,14094)_ = 4.7761, *p* < 2.2 × 10^−16^). ***p < 0.001. ***H***, Distribution (kernel density estimates) of absolute lag values at peak cross-correlation for all sessions across 100% (top) and 50% (bottom) reward conditions for WT and *Fmr1^−/y^* rat pairs. KS test (100%: *p* > 0.05, 50%: *p* < 0.01). ns, not significant, **p < 0.01. ***I***, Session wise peak correlation coefficients for WT and *Fmr1^−/y^* rat pairs, showing greater temporal coordination for WT rat pairs. 100%: Two-way RM ANOVA (genotype: *F*_(1,168)_ = 13.1922, *p* = 0.0003731, session: *F*_(20,168)_ = 0.5387, *p* = 0.9463845, genotype × session: *F*_(20,168)_ = 0.5070, *p* = 0.9610644). 50%: Two-way RM ANOVA (genotype: *F*_(1,280)_ = 18.7522, *p* = 2.075 × 10^−5^, session: *F*_(34,280)_ = 0.7612 *p* = 0.8302, genotype × session: *F*_(34,280)_ = 0.3933, *p* = 0.9992). ***p < 0.001.

Having established that WT and *Fmr1* rats differed in the spatial organization of their joint exploration strategy, we next asked whether they also differed in the temporal precision of their coordination. We first quantified the temporal dynamics of rats’ behavior based on their arrival and departure times at reward wells (Fig. 4*A*). To examine how these behavioral dynamics evolved over training, we quantified three temporal parameters: mean arrival lag between rats during match events (Fig. 4*B*), mean departure lag from wells following matches (Fig. 4*C*), and mean dwell time at wells during both match and non-match events (Fig. 4*D*). We observed that, under the 100% reward contingency, the mean arrival lag during match events progressively decreased in WT pairs, whereas it remained consistently elevated in *Fmr1* pairs (Fig. 4*B*). This difference persisted under the 50% reward contingency, with WT pairs exhibiting significantly shorter arrival lags than *Fmr1* pairs (Fig. 4*B*). A similar pattern emerged for mean departure lag following match events, with WT rats departing more promptly than *Fmr1* rats (Fig. 4*C*). Additionally, *Fmr1* rats exhibited longer dwell times at reward wells compared with WT rats (Fig. 4*D*).

To further probe whether these differences in arrival, departure, and dwell time dynamics reflected broader patterns of temporal coordination, we next examined the fine-grained synchrony of transitions using cross-correlation analyses of arrival times of rats in a pair at reward wells (Fig. 4*E–I*). Cross-correlation of arrival times at reward wells revealed significant peaks at multiple lags for both groups, exceeding shuffled controls and indicating that pairs learned to align their transitions within characteristic temporal windows. Importantly, only WT rats showed prominent peaks near zero lag, reflecting near-synchronous transitions consistent with a gaze-independent strategy that emerged with training, indicating that rats in a pair arrive at reward wells in close temporal proximity, moving in tandem in synchronous movements/trajectories (example in Movie 2). *Fmr1* rats lacked this short-lag synchrony, indicating a reliance on asynchronous movements (example in Movie 1) and possibly a gaze-dependent strategy involving a follower tracking a leader’s position followed by a reactive action to match positions. Correspondingly, the distribution of lags at peak correlation coefficients differed between WT and *Fmr1* rats, particularly under the 50% condition (Fig. 4*H*), and achieved higher peak correlation values across sessions (Fig. 4*I*).

**Movie 2.** Example video showing synchronous transitions of rat pairs. Example trials show near-synchronous transitions of two rats resulting in successful cooperation. It also illustrates the higher order complex predictive matching strategy employed by WT rats. [View online]

Together, these findings demonstrate that WT rats not only structured their exploration in space but also synchronized it in time, enabling efficient joint foraging. *Fmr1* rats showed deficits in both domains. They failed to organize exploration into structured sequences and were unable to coordinate transitions with short temporal lags. Overall, their behavior was organized at longer timescales relative to their WT counterparts. Further, the differences in structured exploration were exclusive to the social context of the cooperative foraging task and not to other non-social contexts as seen in the control experiments. These impairments highlight a specific vulnerability in the social decision-making mechanisms required for cooperative behavior.

### Dynamic turn-taking in the initiation of coordinated responses

**Figure 5.**
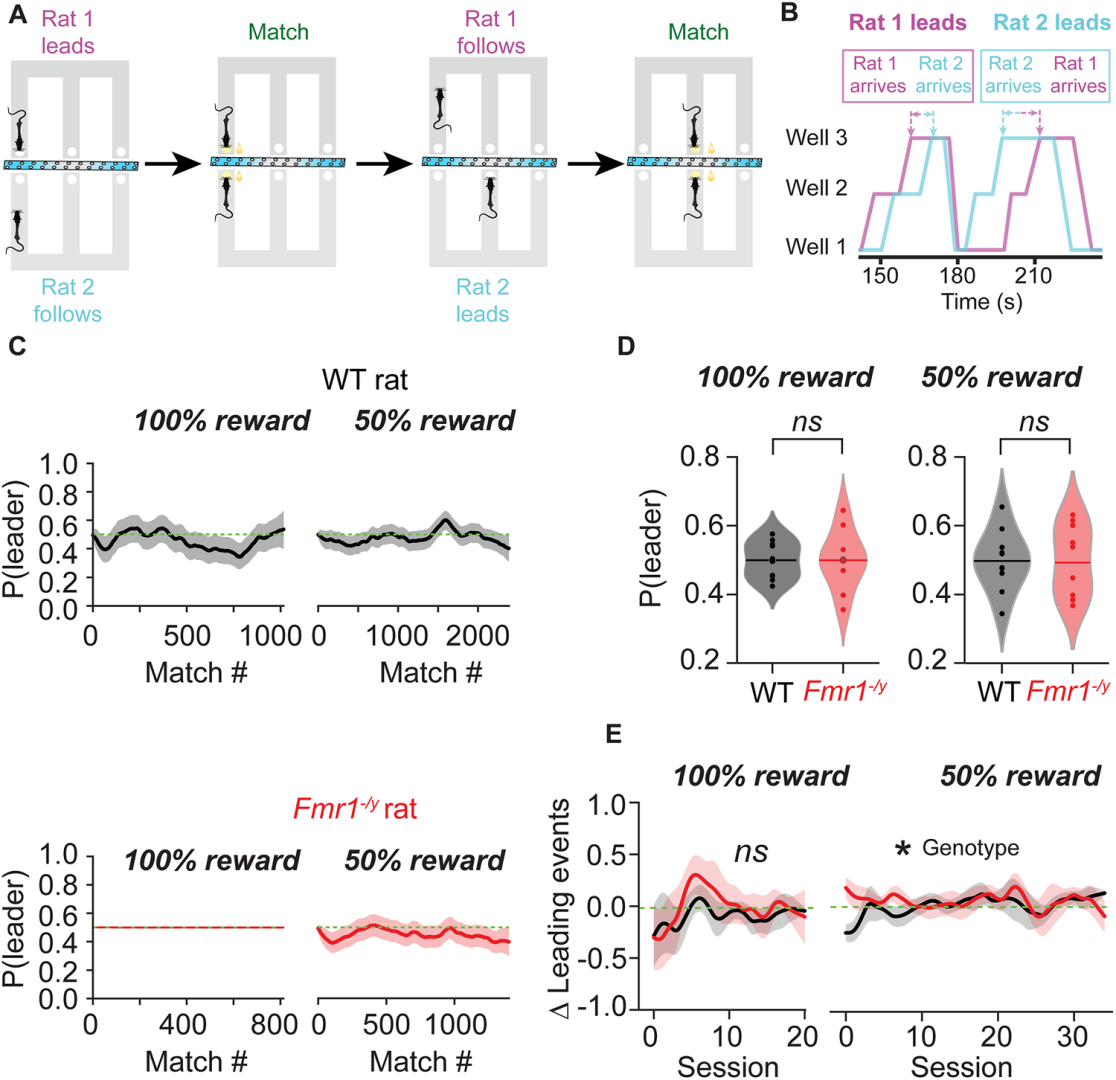
Dynamic leader–follower relationship during cooperative behavior. ***A***, Schematic illustrating dynamic leader–follower role switching between rats within a session. ***B***, Snippet from an example session showing leader–follower relationship between rats in a pair for exemplar match events. ***C***, Probability of assuming leader role for an example WT (top) and *Fmr1^−/y^* rat (bottom) within respective pairs as predicted by a state-space model under 100% (left) and 50% reward conditions (right). Green dashed horizontal line denotes probability of 0.5. ***D***, Mean probability of assuming leader role (state space model) under 100% (left) and 50% (right) reward contingencies (Mann–Whitney *U* test, *p* > 0.05). ns, not significant. ***E***, Difference in proportion of leading events between rats in a pair in 100% (left) and 50% reward contingencies (right). 100%: Two-way RM ANOVA (genotype: *F*_(1,168)_ = 1.6179, *p* = 0.2051, session: *F*_(20,168)_ = 1.0130, *p* = 0.4496, genotype × session: *F*_(20,168)_ = 0.4402, *p* = 0.9825). 50%: Two-way RM ANOVA (genotype: *F*_(1,264)_ = 4.1638, *p* = 0.04229, session: *F*_(32,264)_ = 1.1584, *p* = 0.26302, genotype × session: *F*_(32,264)_ = 1.0370, *p* = 0.41810). ns, not significant, *p < 0.05.

Fixed leader–follower roles are among the most common strategies for organizing coordinated group behavior in social animals (King et al., 2009; Massen et al., 2010; Gachomba et al., 2022; Cheng et al., 2025; Meisner et al., 2025). Clear peaks at both negative and positive lags in the cross-correlation of arrival times prompted us to examine these dynamics more directly, focusing on sequences of coordinated match events across trials (Fig. 5). For each match event, the rat that arrived first at the reward well was designated as the “Leader” and the peer as “Follower” for that event, with similar leader–follower designations for subsequent match events (Fig. 5*A*,*B*). The probability of maintaining the leader role across consecutive match events remained near chance levels for both WT and *Fmr1* rats over the course of learning (Fig. 5*C–E*), indicating flexible and dynamic turn-taking by rats in a pair, i.e., no fixed leader–follower roles were seen (example in Movie 3). On average, both genotypes led approximately half of the match events under both 100% and 50% reward contingencies (Fig. 5*D*,*E*). However, under the 50% reward condition, a small but significant genotype-specific difference emerged, with WT and *Fmr1* rats differing significantly in their likelihood of assuming the leader role at the start of training (Fig. 5*E*). However, this genotype-specific difference disappeared as training progressed. Furthermore, the likelihood of a rat assuming the leader role on a given trial was modulated by its leadership status over the preceding trials, indicating that leadership assignments fluctuate dynamically over time rather than remaining fixed (Fig. S3*C,D*). These findings demonstrate dynamic turn-taking behavior in WT and *Fmr1* rats in assuming leadership roles in this cooperation task.

**Movie 3.** Example video showing dynamic leader–follower relationship between rats in a pair. Example trials showing how rats dynamically exchange roles as leaders while cooperating with each other. [View online]

### Differential reliance on two hierarchical strategies for successful coordination

Peer-directed social attention is a well-established hallmark of cooperative behavior across species, including primates and marmosets (Emery et al., 1997; Nahm et al., 1997; Franch et al., 2024; Meisner et al., 2025). Performance deficits in the sensory-deprived condition of the cooperative foraging task indicated that vision-based social attention was essential for coordinating foraging across WT and *Fmr1* rat pairs. We hypothesized that rats strategically deploy social gaze (quantified using peer-directed head orientation) to gather task-relevant information and facilitate coordination. To examine this, we adopted a multipronged approach combining computational modeling and data-driven analyses.

**Figure 6.**
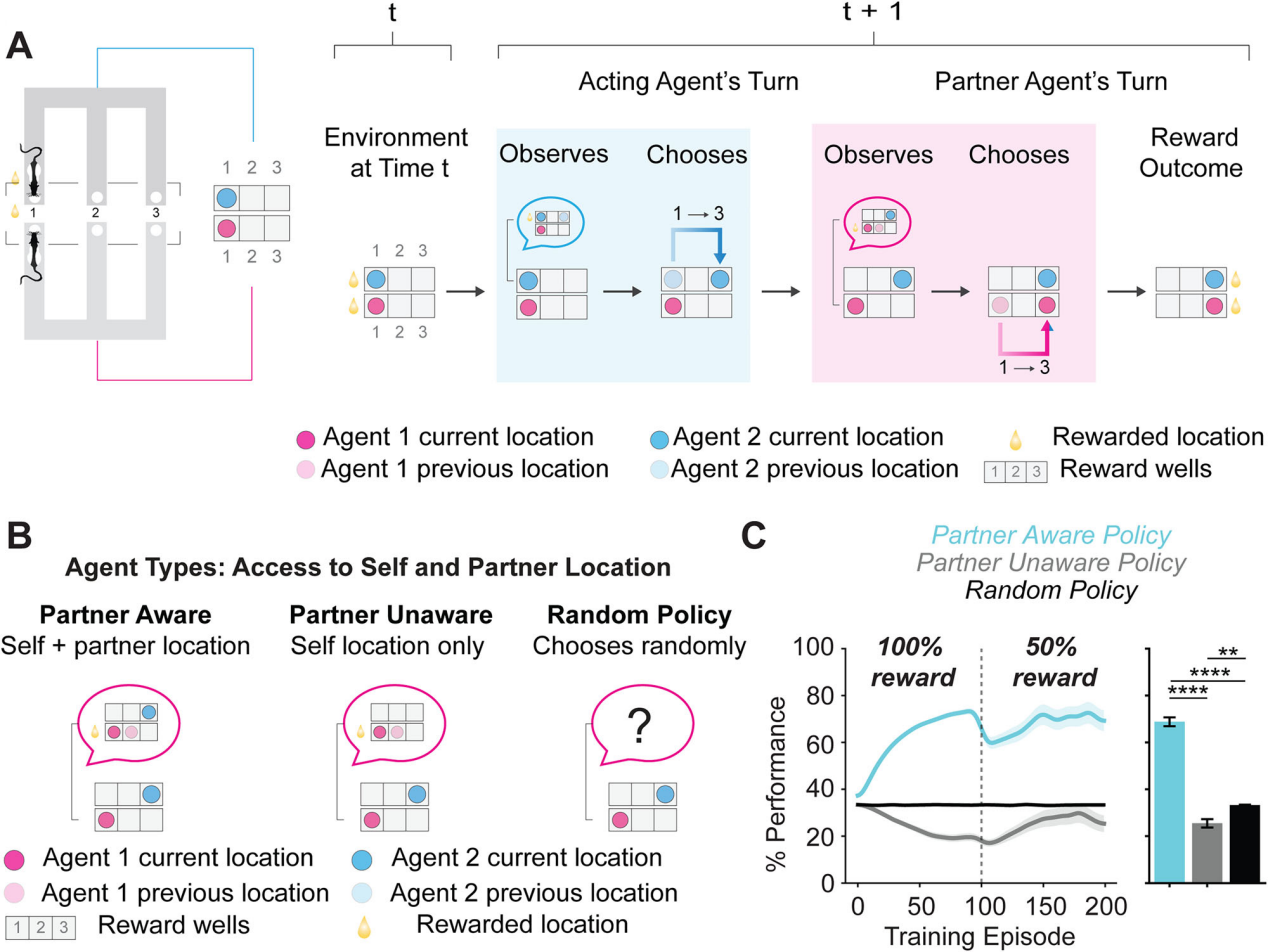
Multiagent reinforcement learning framework reveals partner information is critical for cooperative learning. ***A***, Schematic of the spatial cooperation task (left) and corresponding cooperative multiagent reinforcement learning (MARL) framework (right). Each agent navigates its own discrete three-location grid. On each timestep, one agent is randomly selected to act first. The first-acting agent observes the current environmental state, which includes its current and past locations, last rewarded location, and its partner's current location and then makes a choice. The partner agent subsequently observes the updated state and selects its own action. A joint mutual reward is delivered if both agents choose the same location, provided it differs from the last rewarded location. ***B***, Agent types differ in available task-relevant information. Partner-aware agents observe both their own and their partner's current location. Partner-unaware agents observe only self-related cues. Random agents receive no observations and select among the three locations at random. ***C***, Partner awareness enhances cooperative learning. Learning curves show cooperation efficiency (% of optimal matches) over 200 training episodes for Partner-Aware (cyan), Partner-Unaware (gray), and Random (black) agents. A reward contingency switch at episode 100 (dashed line) reduces the reward probability from 100 to 50%, similar to the spatial coordination task, to test agents’ adaptability under reduced reward certainty. Partner-aware agents rapidly achieve high coordination, reaching ∼70% efficiency post-switch, and significantly outperform both other groups. Partner-unaware agents perform sub-optimally, even compared with random agents. Repeated-measures ANOVA confirmed the main effect of agent type (*F*_(2,198)_ = 225.34, *p* < 0.0001). Post hoc Wilcoxon tests showed that partner-aware agents outperformed both partner-unaware and random agents (*p* < 0.0001). Friedman test further supported this effect (*p* < 0.0001). Right, Final coordination efficiency after training. Partner-aware agents achieved significantly higher performance than both partner-unaware and random agents (*p* < 0.0001). Error bars represent mean ± SEM; statistical comparisons are indicated above.

First, using a multiagent reinforcement learning (MARL) framework (Tan, 1993; Du et al., 2023; Tsutsui et al., 2024), we evaluated the contribution of social attention to cooperative foraging. In these simulations, agent pairs foraged between three reward patches (analogous to the three reward wells in the maze) under three distinct policies: partner-aware (agents received partner-related information alongside environmental and self-related information), partner-unaware (agents received only environmental and self-related information), and random (agents received partner-related information alongside environmental and self-related information but made random choices; Fig. 6*A*). Agents in a pair were required to coordinate their choices similar to those in the cooperative foraging task, with both receiving a discounted reward on successful coordination (Fig. 6*B*). Like rat pairs, agent pairs started their training under 100% reward condition and after 100 training episodes switched to 50% reward condition for another 100 episodes. Partner-aware agent pairs achieved markedly higher performance than both partner-unaware and random agent pairs (Fig. 6*C*), underscoring the critical role of social attention in cooperative foraging.

Building on these model results, we next sought to identify and quantify behavioral markers of peer-directed visual attention in rats performing the cooperative task. To this end, we categorized behavior into two trial types: synchronous trials, in which both rats moved in tandem from one well to another (example in Movie 2), and asynchronous trials (example in Movie 1), in which rats initially occupied different reward wells at the beginning of the trial. The asynchronous configuration permitted, though did not necessitate, visual monitoring of the partner, making it particularly suitable for assessing peer-directed attention.

**Figure 7.**
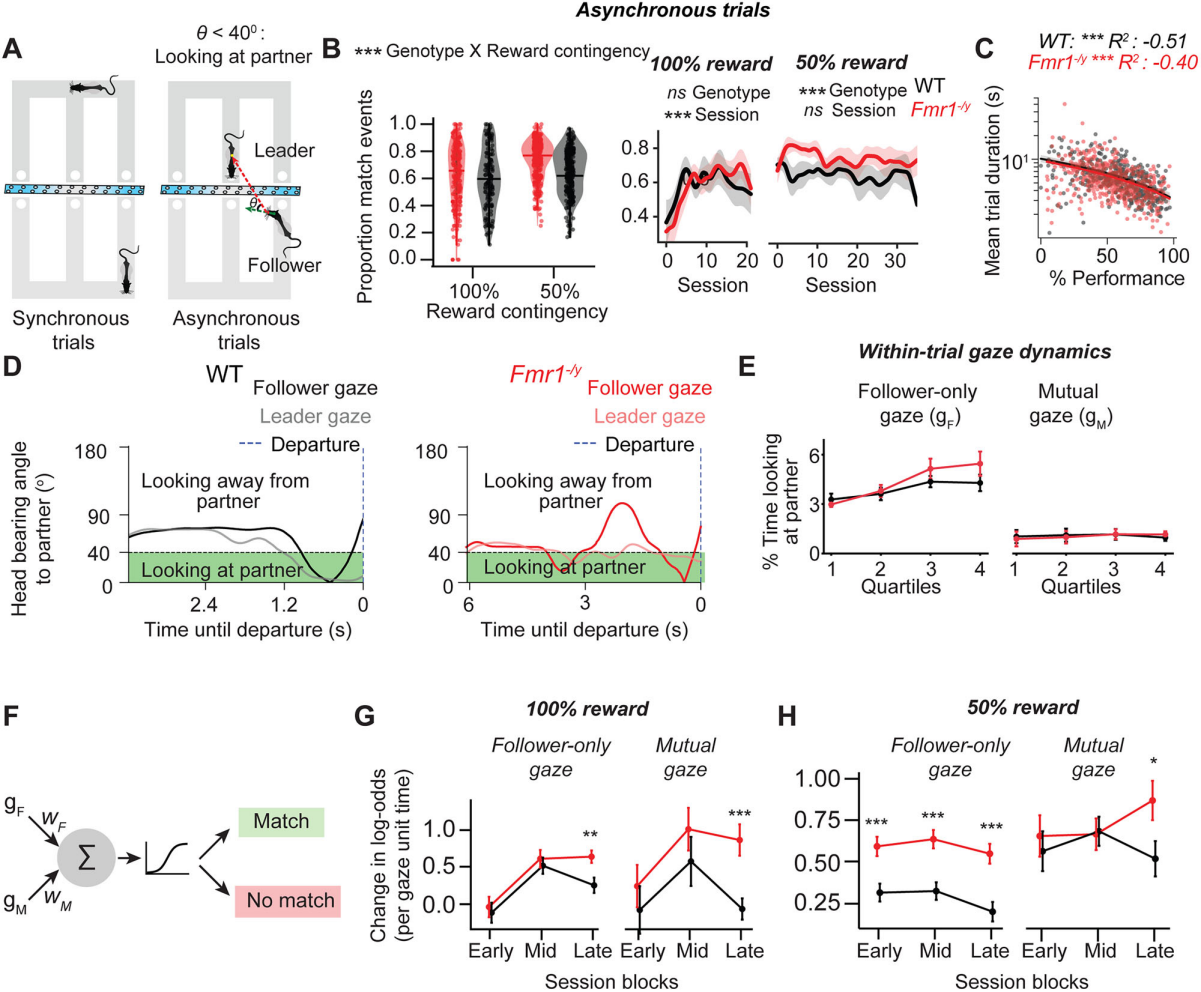
Role of partner-directed attention in successful cooperative behavior. ***A***, Schematic showing synchronous and asynchronous trial type assignments and quantification of head bearing angles toward partner rats during asynchronous trials. ***B***, Comparison of proportions of match events for WT and *Fmr1^−/y^* rats that occurred in asynchronous trials (left); session-wise comparison of proportion of match events occurring in asynchronous trials (right; 100%: Two-way RM ANOVA, genotype: *F*_(1,168)_ = 1.3859, *p* = 0.2407586, session: *F*_(20,168)_ = 2.4594, *p* = 0.0009798, genotype × session: *F*_(20,168)_ = 0.7050, *p* = 0.8175007. 50%: Mixed-effects linear model, genotype: *F*_(1,280)_ = 26.002, *p* = 6.292 × 10^−7^, session: *F*_(34,280)_ = 0.4583, *p* = 0.9962, genotype × session: *F*_(34,280)_ = 0.4583, *p* = 0.9962). ***p < 0.001. ***C***, Pearson’s correlation between mean trial duration and performance for each session (combined across 100% and 50% reward contingencies). WT: *R* = −0.51, *p* = 2.79 × 10^−29^; *Fmr1^−/y^*: *R* = −0.40, *p* = 5.11 × 10^−8^. ***p < 0.001. ***D***, Example trials showing follower and leader gaze dynamics for a WT (top) and *Fmr1^−/y^* (bottom) rat. Shaded green area represents when head bearing angle falls below threshold of 40°. ***E***, Within-trial dynamics of gaze variables. A mixed-effects ANOVA revealed significant effects of quartile for follower-only gaze (*F*_(3,54)_ = 11.77, *p* < 0.001, $\eta_{p}^{2}=0.40$), but not for mutual gaze (*F*_(3,54)_ = 0.56, *p* > 0.05). Post hoc comparisons (Holm-corrected) showed that follower-only gaze duration increased from quartile 1 through quartiles 2, 3, and 4 (*p* < 0.05). No significant effects of genotype or interactions were observed. ***F***, Generalized linear mixed-effects model (GLMM) for assessing effects of gaze predictors on successful coordination (see Materials and Methods for details). ***G***, Post hoc comparison (Tukey-adjusted) of effects of different gaze variables during different phases of training on change in log-odds of matching for WT (black) and *Fmr1^−/y^* (red) rats for 100% reward condition. Follower-only gaze: Early: contrast (*Fmr1^−/y^* - WT) estimate = −0.03 ± 0.21, *p* = 0.89; Mid: contrast (*Fmr1^−/y^* - WT) estimate = 0.03 ± 0.18, *p* = 0.87; Late: contrast (*Fmr1^−/y^* - WT) estimate = 0.34 ± 0.15, *p* = 0.02. Mutual gaze: Early: contrast (*Fmr1^−/y^*- WT) estimate = −0.23 ± 0.48, *p* = 0.63; Mid: contrast (*Fmr1^−/y^* - WT) estimate = −0.17 ± 0.51, *p* = 0.73; Late: contrast (*Fmr1^−/y^* - WT) estimate = 0.60 ± 0.28, *p* = 0.03. **p < 0.01, ***p < 0.001. ***H***, Same as ***G***, but for 50% reward condition. Follower-only gaze: Early: contrast (*Fmr1^−/y^* - WT) estimate = 0.38 ± 0.10, *p* = 4.28 × 10^−5^; Mid: contrast (*Fmr1^−/y^* - WT) estimate = 0.39 ± 0.09, *p* = 6.76 × 10^−6^; Late: contrast (*Fmr1^−/y^* - WT) estimate = 0.44 ± 0.10, *p* = 4.96 × 10^−6^. Mutual gaze: Early: contrast (*Fmr1^−/^*^y^ - WT) estimate = 0.04 ± 0.20, *p* = 0.83; Mid: contrast (*Fmr1^−/y^* - WT) estimate = −0.02 ± 0.15, *p* = 0.87; *Late:* contrast (*Fmr1^−/y^* - WT) estimate = 0.20 ± 0.18, *p* = 0.28. *p < 0.05, ***p < 0.001.

We therefore focused on asynchronous trials for quantifying peer-directed attention. We began by examining the distribution of cooperative matches across trial types. In WT pairs, matches were more evenly distributed between synchronous and asynchronous trials, whereas *Fmr1* pairs showed a higher proportion of matches occurring during asynchronous trials, particularly under the 50% reward condition (Fig. 7*A*,*B*). This indicates that WT matches occurred across both trial contexts, while *Fmr1* matches were more concentrated in asynchronous trials. Examining how asynchronous versus synchronous match proportions evolved over training, we found that under the 100% reward condition, both genotypes showed a steady increase in match proportions of asynchronous trials which then remained stable across sessions. In contrast, under the 50% reward condition, a clear genotype-specific difference emerged, with *Fmr1* pairs showing a distinct pattern, with higher usage on asynchronous trails for match events compared with WT pairs (Fig. 7*B*). Next, we found that session-level mean trial duration was negatively correlated with cooperative performance, indicating that shorter, more efficient trials were associated with higher performance (Fig. 7*C*).

We then focused on specific social gaze variables and their contribution to cooperative decisions. We quantified head orientation to estimate for gaze behavior. Head orientation vectors were derived from head-to-neck tracking, and a 40° angular threshold relative to the line of sight was applied to determine whether the partner was within the rat’s binocular field of view (Wallace et al., 2013; Fig. 7*A*, example in Movie 4). Two gaze types were quantified for each trial: follower-only gaze (follower oriented toward leader) and mutual gaze (both rats simultaneously oriented toward each other). Durations of these gaze events were normalized relative to total trial duration. We began by characterizing when rats directed their gaze toward their partners during the task. Across genotypes, partner-directed gaze occurred most frequently in the final quartile of a trial, just prior to departure (Fig. 7*D*,*E*), indicating that visual monitoring was closely tied to movement initiation.

**Movie 4.** Example video showing quantification of rats' peer-directed head orientation event as estimated from tracked keypoints. This example trial demonstrates the approach used to identify and quantify peer-directed head orientation events based on keypoint tracking of rat body parts. [View online]

Finally, to formally test how different forms of social gaze contributed to cooperative outcomes, we fit generalized linear mixed-effects models (GLMMs) with genotype, training block, and normalized gaze durations as predictors of trial-level cooperation (Fig. 7*F*), analyzed separately for these asynchronous trials under 100% and 50% reward contingencies (Fig. 7*G*,*H*). In our coding scheme, follower-only gaze indicates trials in which the follower oriented toward the leader (but not vice versa), whereas mutual gaze indicates reciprocal orientation by both rats. Under the 100% reward condition, the models revealed that follower-only gaze became an increasingly strong predictor of successful cooperation across training (Fig. 7*G*, left; Table S1). Post hoc comparisons indicated that this effect was driven primarily by *Fmr1* pairs. They continued to rely heavily on the follower attending to the leader to coordinate successfully. In contrast, WT pairs showed a marked reduction in reliance on follower gaze by the final training block, suggesting that coordination became more efficient and less dependent on explicit visual monitoring. A similar pattern was observed for mutual gaze (Fig. 7*G*, right), where the initial reliance diminished over training, consistent with WT pairs transitioning from gaze-dependent to more automatic strategies.

Under the 50% reward condition (Fig. 7*H*, Table S2), both follower-only and mutual gaze significantly increased the likelihood of coordination overall. However, their use was modulated by genotype and training stage. *Fmr1* pairs remained significantly more dependent on follower-only gaze, i.e., the follower monitoring the leader throughout, compared with WT pairs (Fig. 7*H*, left). For mutual gaze (Fig. 7*H*, right), both genotypes initially benefited from reciprocal monitoring, but this effect declined in later training blocks for WT pairs, again consistent with a shift toward gaze-independent coordination strategies once task contingencies were learned.

Together, these results demonstrate that social gaze in rats, like primates (Meisner et al., 2025), plays a central role in shaping cooperative outcomes but that its contribution diverges sharply across genotypes and with training progression. Computational modeling established that partner-aware strategies confer a distinct advantage in cooperative foraging, underscoring the adaptive value of social attention. Behavioral analyses confirmed that rats engaged in peer-directed gaze during asynchronous trials. However, while *Fmr1* rats remained consistently reliant on gaze-dependent strategies across training, WT rats exhibited an adaptive shift. They initially deployed gaze as a tool for scaffolding coordination, but progressively transitioned toward a gaze-independent, temporally synchronized strategy. This flexible reduction in gaze reliance highlights an adaptive reorganization of coordination strategies in WT rats, a process that does not emerge in *Fmr1* rats.

### Partner cooperation history shapes cooperative behavior

**Figure 8.**
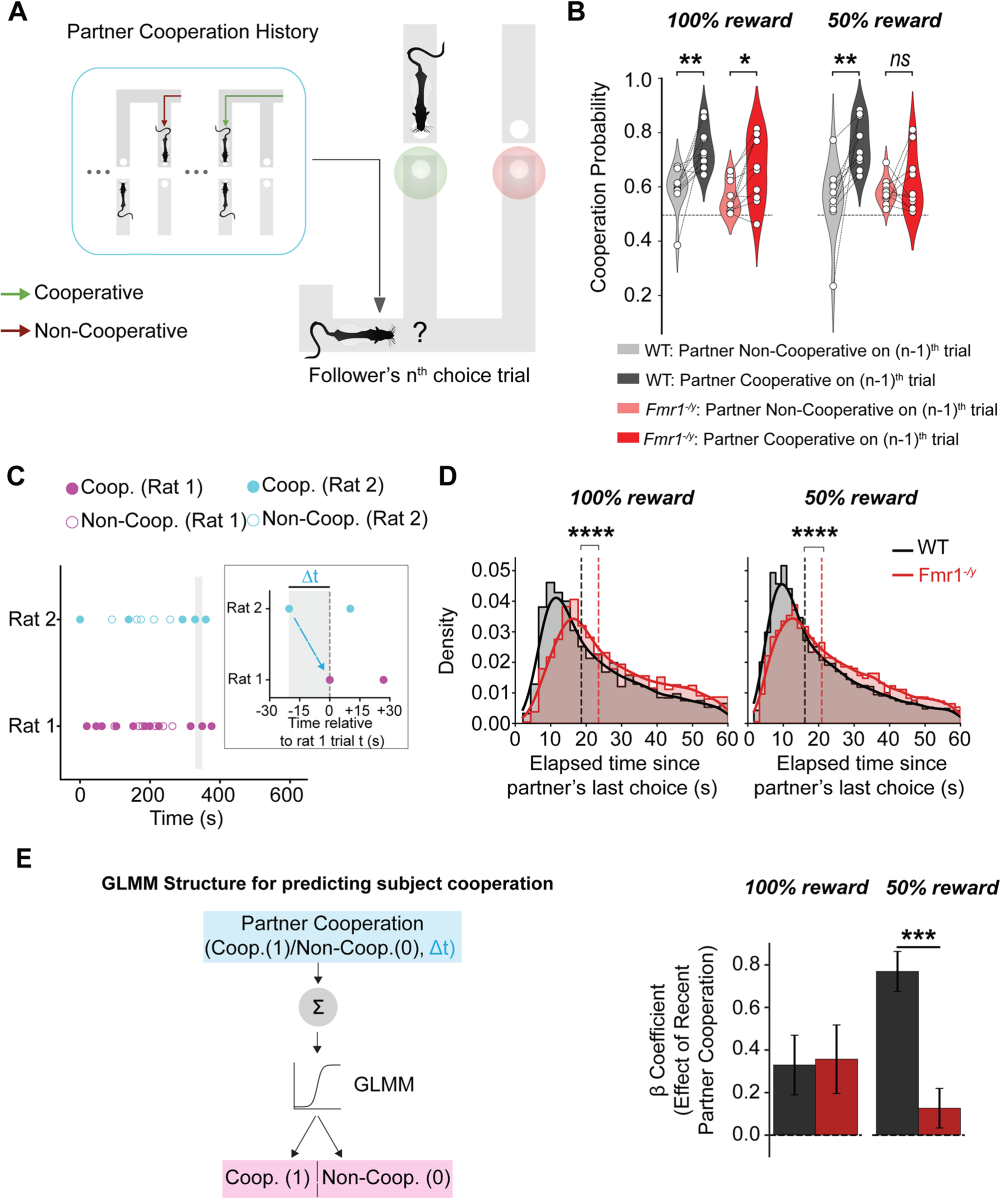
Recent partner choices influence cooperation in WT but not *Fmr1^−/y^* rats. ***A***, Schematic of a follower trial. On each trial, one rat (leader) transitions between the arms first; then the other (follower) decides whether to cooperate. ***B***, Cooperation probability as a function of the partner’s prior choice in WT and *Fmr1^−/y^* rats under 100% and 50% reward contingencies. Wilcoxon signed-rank test: WT rats, 100%: *p* < 0.01; 50%: *p* < 0.01. *Fmr1^−/y^* rats, 100%: *p* < 0.05; 50%: *p* > 0.05. **p < 0.01, *p < 0.05. ns, not significant. ***C***, Timeline of cooperative (filled) and noncooperative (open) follower choices made by Rat 1 (magenta) and Rat 2 (cyan) in a representative session from a single pair. Inset, Zoomed view of a follower trial from Rat 1, showing the most recent cooperative follower choice by Rat 2. Δ*t* denotes the time lag between the rat’s choices. ***D***, Distributions show the elapsed time (Δ*t*) between the partner’s most recent choice and the subject’s current trial under the 100% (left) and 50% (right) reward contingencies. Curves are truncated at 60 s to highlight behaviorally relevant timescales. Dashed lines indicate genotype-specific medians. Across both contingencies, *Fmr1^−/y^* rats showed significantly longer partner-referenced Δ*t* intervals than WT rats (100%: *U* = 6,515,700, *p* < 0.0001; 50%: *U* = 4.25 × 106, *p* < 0.0001). These timing differences justify the inclusion of Δ*t* and its interactions as covariates in the GLMM to account for baseline differences in decision timing between genotypes. ***E***, GLMM estimating rats' cooperation based on partner's previous choice, Δ*t*, and genotype. WT rats show significantly higher contribution of recent partner cooperation in the 50% condition. Fixed-effect *β* coefficients (±95% CI): WT rats, 100%: *β* = 0.36, *p* < 0.001; 50%: *β* = 0.77, *p* < 0.001. *Fmr1^−/y^* rats, 100%: *β* = 0.26, *p* < 0.01; 50%: *β* = 0.13, *p* < 0.01. Genotype × partner cooperation interaction at 50%: *β* = 0.64, *p* < 0.001. *p < 0.001, *p* < 0.01, *p* < 0.05.

Social decision-making requires monitoring and adapting to others' recent actions (Haroush and Williams, 2015; Báez-Mendoza et al., 2021). To test whether rats use partner behavior to guide cooperation, and whether this is disrupted in *Fmr1* rats, we analyzed trial-by-trial choices across the two reward conditions (Fig. 8*A*,*B*). For the 100% reward contingency, both WT and *Fmr1* rats were more likely to cooperate after their partner had just done so in the previous trial (Fig. 8*B*). In contrast, for the 50% reward contingency, only WT rats adjusted their behavior based on recent partner choices.

To formally assess how partner behavior influenced cooperation, we fit a generalized linear mixed-effects model (GLMM) predicting the likelihood of cooperation on each trial based on the partner's previous choice, the elapsed time since that choice (Δ*t*), and their interaction (Fig. 8*C*). Including Δ*t* accounted for genotype-related differences in trial timing, such as longer inter-choice intervals in *Fmr1* rats (Fig. 8*D*), allowing us to isolate the specific contribution of partner behavior. To control for individual tendencies, we also included predictors reflecting each rat’s own recent choices. Genotype was included as an interaction term, and random intercepts for rat and session accounted for repeated measures. The GLMM model confirmed that both WT and *Fmr1* rats were more likely to cooperate following a cooperative partner choice under the 100% reward contingency. However, under the 50% contingency only WT rats adjusted their choices based on recent partner behavior, while *Fmr1* rats did not (Fig. 8*E*).

Together, these findings suggest that WT rats flexibly adjust cooperative behavior in response to recent partner actions, by adapting coordination strategies together with their partner as a unit. In contrast, *Fmr1* rats continue to rely primarily on the less flexible follower tracking strategy of lower efficiency, with weaker associations with partner actions on the immediately preceding trial, leading to significantly deficient performance especially in the probabilistic reward condition.

## Discussion

In this study, we developed a novel rat spatial cooperative behavior paradigm to investigate social coordination strategies that enable successful cooperation. This design, based on the ethologically relevant framework of spatial foraging, enables continuous social decision-making in rat dyads under controlled settings, including in a rat model of Fragile X syndrome. We characterized peer coordination underlying cooperative behavior and investigated how these processes are disrupted in *Fmr1* rat pairs. We found that both WT and *Fmr1* rat pairs were able to coordinate cooperative visits to the matching reward wells; however, WT rats were more efficient by employing flexible strategies that utilized knowledge and anticipation of partner choice patterns. *Fmr1* rats relied heavily on a sequential, reactive strategy that involved monitoring partner choices as followers and then executing actions to match partner location, a slower approach that requires asynchronous choices by rats in a pair. In contrast, WT pairs also utilized knowledge of the task structure by spatially and temporally coordinating choice sequence patterns with their partner, a predictive strategy that enabled faster, synchronous transitions, especially under the probabilistic reward condition. Thus, WT rats employ a complex higher order social coordination strategy for efficient cooperation, especially under challenging probabilistic conditions, which is deficient in *Fmr1* rats.

Our task differs from previous rodent cooperative foraging paradigms (Avital et al., 2016; Jiang et al., 2021, 2026; Zhang et al., 2023; Cheng et al., 2025; Erlich et al., 2025) in several aspects. First, rats in a pair occupied separate mazes, rather than sharing the same environment, offering several advantages for studying cooperative behavior. Eliminating direct physical interference ensured that coordination arose from genuine monitoring of the partner's actions rather than from avoidance or displacement. Spatial separation also minimized confounding olfactory cues within mazes, which rats could otherwise use to guide behavior, and distinct mazes enabled precise parametric manipulations of task conditions, such as reward probability or timing, facilitating a clearer assessment of how one animal's behavior influences the other. Collectively, these design features strengthen the interpretation that observed coordination reflects strategic, predictive, and socially mediated decision-making rather than simple responses to shared environmental cues. The use of separate mazes will also enable future investigation of unambiguous neural correlates of partner locations and actions. Further, reward locations were dynamic across three possibilities rather than fixed, preventing animals from relying on extraneous, task-unrelated cues and instead requiring them to continuously update their behavior in response to their partner's behavior in real time.

While both WT and *Fmr1* pairs learned to coordinate above chance levels, WT pairs outperformed their *Fmr1* counterparts at both 100% and 50% reward contingencies. Thus, the efficiency and robustness of cooperative behavior diverged across genotypes, suggesting that the capacity to flexibly adapt coordination strategies is compromised in *Fmr1* rats. This aligns with evidence that Fragile X syndrome is associated with impairments in executive function and social adaptability (Budimirovic et al., 2006; Klaiman et al., 2014; Hooper et al., 2018; Schmitt et al., 2019; Cregenzán-Royo et al., 2022), which may hinder the ability to optimize coordination. The persistence of coordination in *Fmr1* pairs above chance, however, indicates that basic social engagement mechanisms remain intact but are insufficient to achieve the same level of efficiency as WT pairs. These results underscore the translational value of our cooperative foraging paradigm for uncovering subtle, context-dependent social decision-making deficits relevant to neurodevelopmental disorders.

To ensure that the observed differences in coordination efficiency were not confounded by basic sensory or associative deficits, we tested both genotypes on a visual cue-association task. Performance accuracy was comparable, indicating that both groups were able to perceive visual stimuli and associate them with reward locations. *Fmr1* rats did exhibit slower response latencies in the visual association task relative to WT rats, which parallels the broader organization of cooperative foraging behavior in *Fmr1* rats, which unfolded at longer temporal scales compared with WT rats.

A sequential mode of decision-making, where delaying one's own actions facilitates incorporating information about a partner's choice, has been shown to promote coordination (Rapoport, 1997; Brocas et al., 2018; Moeller et al., 2023). Here, rats initially relied on such sequential strategies, exhibiting non-zero arrival and departure lags at reward wells relative to their partners. These temporal offsets provided a scaffold for establishing cooperative foraging. As training progressed, WT rats also developed a flexible mixture of sequential and simultaneous strategies, reflected in reduced lags and the emergence of near-synchronous transitions seen in cross-correlation analyses. In contrast, *Fmr1* rats failed to show comparable flexibility, maintaining elevated lags and lacking short-lag synchrony, suggesting a persistent reliance on sequential strategies without the adaptive shift toward simultaneous coordination that supports efficient cooperation. This difference in utilization of a simpler, asynchronous strategy, as against a complex, predictive, and synchronous strategy indicates a hierarchical organization of strategies, with efficient cooperation requiring anticipation and prediction of partner actions for synchronization. The use of diverse strategies by WT rat dyads aligns with similar recent reports in a marmoset cooperative lever-pressing task (Meisner et al., 2025). It is possible that the flexible, predictive coordination strategy that we describe here in rat dyads is a hallmark of advanced socio-cognitive abilities.

Furthermore, we observed a dynamic emergence of leader–follower patterns rather than a fixed pattern, which arise naturally from the demands of coordination and are particularly prominent in species where social and ecological pressures favor acting together (Krause and Ruxton, 2002). The dynamic leader–follower interactions observed may reflect a form of spontaneous turn-taking, similar to patterns reported in humans (Clavien and Chapuisat, 2016; Moeller et al., 2023). In human dyads, alternating leadership roles facilitates coordination by allowing individuals to anticipate their partner's actions, maintain joint performance, and avoid conflicts over decisions (Moeller et al., 2023). In our task, rats could potentially use a similar strategy as the reward structure did not confer any advantage to the leader in terms of reward acquisition. While both WT and *Fmr1* pairs exhibited these dynamic patterns, the benefits of such turn-taking may become particularly apparent in contexts requiring rapid, continuous adjustments.

The sensory-deprived (SD) control experiments, in which rat pairs were required to coordinate without visual access to one another, underscored the essential role of social vision in sustaining coordination. This finding was reinforced by computational modeling: partner-aware agents, those with access to information about their partner in addition to self and environmental cues, achieved markedly higher coordination efficiency than agents operating under partner-unaware random policies. Complementary analyses of head direction-based gaze measures further revealed that gaze was especially important for establishing coordination during the early and middle stages of training. By late training blocks when performance was optimized, WT rats shifted from a primarily gaze-dependent strategy to a more gaze-independent mode, consistent with the emergence of predictive coordination strategies.

The gaze-independent mode depends on maintaining an internal model of current partner strategy and timing, resulting in higher efficiency, and is in marked alignment with similar reports in a marmoset cooperation task (Meisner et al., 2025). In this framework, animals in dyads can continue to operate in a coordinated choice pattern regime without requiring continuous monitoring of partner actions, which will only be required to confirm or update the choice patterns and timing in the event of occasional failures. Accordingly, trial duration was negatively correlated with performance (Fig. 7*C*). In contrast, *Fmr1* rats continued to rely heavily on gaze, indicating a reduced capacity to transition from reactive, visually driven strategies to higher-level predictive control. This is also apparent from the analysis (Fig. 8) that shows that partner cooperation history is a significant predictor of ongoing coordination only in WT rats but not in *Fmr1* rats. This persistent reliance highlights a broader deficit in cognitive flexibility and abstraction that may limit their adaptability in social contexts.

In support, WT rats displayed higher-order spatial and temporal coordination strategies as training progressed. Their choice sequences became increasingly structured (lower entropy), with a marked rise in the frequency of optimal triplets and the emergence of near-synchronous transitions, all of which paralleled their reduced reliance on gaze. *Fmr1* rats developed these strategies to a significantly lesser extent, remaining reliant on gaze-based coordination throughout the task. The failure of *Fmr1* rats to make this shift to more efficient predictive strategies based on internal models of their partner's behavior points to a broader difficulty in transitioning from externally monitored, effortful strategies to automatic, internalized routines of social coordination.

Recent work in nonhuman primates has identified the emergence of higher-order, temporally synchronized coordination strategies that develop with extended training (Haroush and Williams, 2015; Meisner et al., 2025). These strategies likely involve internal models of a partner's actions, an advanced form of social cognition that enables flexible, predictive coordination. Our task provides evidence for such higher-order, predictive socio-cognitive strategies in rats. This study thus establishes a powerful tractable rodent model for assessing social interactions and provides a foundation to investigate the neural basis of social representations, reciprocal interactions, strategies underlying complex social behavior, and how impairments in these processes can affect social behavior in animal models of autism spectrum disorders.
